# Supplementary material for: Regulating vesicle bilayer permeability and selectivity via stimuli-triggered polymersome-to-PICsome transition
Source: Nat Commun. 2020 Mar 23;11:1524. doi: 10.1038/s41467-020-15304-x (PMC7090076; doi:10.1038/s41467-020-15304-x)
Supplement: Supplementary file 1 — Supplementary Information [file 41467_2020_15304_MOESM1_ESM.pdf]

Supplementary Information

Regulating vesicle bilayer permeability and selectivity via stimuli-triggered polymersome-to-PICsome transition

Wang *et al.*

Supplementary Methods

Materials.

4-Formylbenzoic acid, 4-mercaptobenzyl alcohol, benzyl mercaptan, 4-(hydroxymethyl)benzoic acid, 2-nitrobenzyl alcohol, 4-dimethylaminopyridine (DMAP), and *N,N′*-dicyclohexylcarbodiimide (DCC) were purchased from Energy Chemical Co., Ltd. *N,N*-Diisopropylaminoethanol, 2-(diethylamino)ethanol, 2-isocyanatoethyl methacrylate (stabilized with BHT), and $NaBH_4$ were purchased from TCI. Dibutyltin dilaurate (DBTL) was purchased from Shanghai Haiqu Chemical Co., Ltd. 2,2-Azoisobutyronitrile (AIBN) was purified by recrystallization from 95% ethanol just prior to use. 2′-Deoxy-5-fluorouridine (5-Fu) and gemcitabine hydrochloride were purchased from Sigma-Aldrich. Doxorubicin hydrochloride (Dox·HCl) was purchased from Iffect Chemphar Co., Ltd. Coumarin 343, Nile red, and calcein were Purchased from J&K Scientific Co., Ltd. Water was deionized with a Milli-Q SP reagent water system (Millipore) to a specific resistivity of 18.4 MΩ·cm. Poly(ethylene glycol)$_{45}$-based macroRAFT agent (PEO$_{45}$-CTA) was synthesized according to previous literature reports.[1,2] Nile red monomer, Nile red-MA, was synthesized according to our previous work.[3] All other reagents were purchased from Sinopharm Chemical Reagent Co., Ltd. and used as received.

Sample Synthesis.

Synthesis of 2-Nitrobenzyl Ester-Photocaged Carboxyl Monomer (NCMA, Supplementary Figure 1a). 2-Nitrobenzyl alcohol (3.0 g, 19.5 mmol, 1.0 equiv.) and 4-formylbenzoic acid (3.21 g, 21.5 mmol, 1.1 equiv.) were dissolved in dry THF (60 mL). DCC (4.83 g, 23.4 mmol, 1.2 equiv.) and DMAP (0.12 g, 0.98 mmol, 0.05 equiv.) were then added. The mixture was stirred at room temperature and the reaction progress was monitored by TLC. After removing all the solvents, the residues were dissolved in dichloromethane (DCM) and extracted with saturated brine. The organic phase was dried over anhydrous $MgSO_4$, filtered, and evaporated to dryness under reduced pressure. The crude product was further purified by flash column chromatography on alkaline aluminum oxide using DCM as the eluent, affording **1** as a solid (5.17 g, yield: 93%). ^{1}H NMR ($CDCl_3$, 300 MHz, Supplementary Figure 6a): δ

10.12 (s, 1H), 8.26 (d, J = 8.19 Hz, 2H), 8.17 (d, J = 8.04 Hz, 1H), 8.00 (d, J = 8.28 Hz, 2H), 7.69 (d, J = 3.30 Hz, 2H), 7.59 (m, 1H), 5.81 (s, 2H).

Next, **1** (4.50 g, 15.8 mmol, 1.0 equiv.) was dissolved in methanol (30 mL), the solution was cooled to 0 ^{o}C in an ice-water bath. $NaBH_4$ (0.60 g, 15.8 mmol, 1.0 equiv.) was added over six aliquots. After the reaction is completed as monitored by TLC, all the solvents were removed under reduced pressure; the residues were dissolved in CH_2Cl_2, and the mixture was washed with saturated NaCl solution, dried over anhydrous $MgSO_4$, filtered, and evaporated to dryness under reduced pressure. The crude product was further purified by flash column chromatography on silica gel using DCM as the eluent, affording **2** as a solid (4.35 g, yield: 96%). ^{1}H NMR ($CDCl_3$, 300 MHz, Supplementary Figure 6b): δ 8.15 (d, J = 8.25 Hz, 1H), 8.10 (d, J = 8.13 Hz, 2H), 7.67 (d, J = 3.06 Hz, 2H), 7.51 (m, 3H), 5.78 (s, 2H), 4.80 (s, 2H).

Finally, **2** (4.00 g, 13.9 mmol, 1.0 equiv.) and 2-isocyanatoethyl methacrylate (2.60 g, 16.7 mmol, 1.2 equiv.) were dissolved in anhydrous DCM (40 mL), catalytic amount of DBTL (50 μL) was added. The reaction mixture was stirred at room temperature and monitored by TLC until the reaction was completed after ~5 h. The crude product was washed with saturated brine, dried over anhydrous $MgSO_4$, filtered, and then evaporated to dryness under reduced pressure. The crude product was further purified by flash column chromatography on alkaline aluminum oxide using DCM as the eluent, affording NCMA monomer as a white solid (5.47 g, yield: 89%). ^{1}H NMR ($CDCl_3$, δ, ppm, Supplementary Figure 7a): 8.15 (d, J = 7.92 Hz, 1H), 8.09 (d, J = 8.19, 2H), 7.67 (d, J = 4.08 Hz, 2H), 7.54 (m, 1H), 7.45 (d, J = 8.19 Hz, 2H), 6.11 (s, 1H), 5.78 (s, 2H), 5.59 (s, 1H), 5.17 (s, 2H), 5.11 (s, 1H), 4.25 (t, J = 10.44, 2H), 3.54 (t, J = 16.47 Hz, 2H), 1.94 (s, 3H). ^{13}C NMR ($CDCl_3$, δ, ppm, Supplementary Figure 7b): 167.62, 165.91, 155.78, 147.48, 142.00, 135.62, 134.08, 132.66, 130.30, 129.04, 128.00, 126.05, 125.44, 65.91, 63.62, 40.50, 18.22.

Synthesis of Disulfide-Caged Carboxyl Monomer (DCMA, Supplementary Figure 1b). 4-(Hydroxymethyl)benzoic acid (4.00 g, 26.3 mmol, 1.0 equiv.) and 2-isocyanatoethyl methacrylate (4.08 g, 26.3 mmol, 1.0 equiv.) were dissolved in anhydrous THF (80 mL), with the subsequent addition of catalytic amount of DBTL (50 μL). The reaction mixture was stirred at room temperature and monitored by TLC until the reaction was completed after ~6

h. The crude product was washed with saturated brine, dried over anhydrous $MgSO_4$, filtered, and then evaporated to dryness under reduced pressure. The crude product was further purified by silica gel column chromatography using DCM as the eluent, affording **3** as a solid (6.70 g, yield: 83%). ^{1}H NMR ($CDCl_3$, *δ*, ppm, Supplementary Figure 8a): 8.10 (d, J = 8.16 Hz, 2H), 7.46 (d, J = 8.13, 2H), 6.98 (s, 1H), 6.12 (s, 1H), 5.59 (s, 1H), 5.18 (t, J = 33.72, 2H), 4.26 (t, J = 10.50, 2H), 3.55 (t, J = 16.80, 2H), 1.94 (s, 3H).

Next, 4-mercaptobenzyl alcohol (5.0 g, 35.7 mmol, 1.0 equiv.) and benzyl mercaptan (4.43 g, 35.7 mmol, 1.0 equiv.) were dissolved in $CHCl_3$ (40 mL) and stirred at room temperature for 30 min. Iodine (13.6 g, 53.55 mmol, 1.5 equiv.) was then added over six aliquots. After 2 h, the mixture was washed with aqueous solution of sodium thiosulfate and saturated brine, dried over anhydrous $MgSO_4$, filtered, and evaporated to dryness under reduced pressure. The crude product was further purified by silica gel column chromatography using DCM/*n*-hexane as the eluent, affording **4** as a solid (3.37 g, yield: 36%). ^{1}H NMR (DMSO-d_6, *δ*, ppm, Supplementary Figure 8b): 7.49 (d, J = 8.22 Hz, 2H), 7.43-7.27 (m, 7H), 4.49 (s, 2H), 4.03 (s, 2H), 3.73 (s, 1H).

Finally, **3** (3.0 g, 9.77 mmol, 1.0 equiv.) and **4** (2.56 g, 9.77 mmol, 1.0 equiv.) were dissolved in anhydrous THF (60 mL), DCC (2.42 g, 11.72 mmol, 1.2 equiv.), DMAP (0.06 g, 0.49 mmol, 0.05 equiv.) was then added. The reaction mixture was stirred at room temperature and monitored by TLC until the reaction was completed after ~8 h. The crude product was washed with saturated brine, dried over anhydrous $MgSO_4$, filtered, and then evaporated to dryness under reduced pressure. The crude product was further purified by flash column chromatography on alkaline aluminum oxide using DCM as the eluent, affording DCMA monomer as a yellowish solid (4.68 g, yield: 87%). ^{1}H NMR (DMSO-d_6, 300 MHz, Supplementary Figure 9a): *δ* 7.97 (d, J = 8.19, 2H), 7.47 (s, 1H), 7.38 (d, J = 5.64 Hz, 2H), 7.34-7.22 (m, 9H), 6.03 (s, 1H), 5.64 (s, 1H), 5.33 (d, J = 11.37 Hz, 2H), 5.11 (s, 2H), 4.25 (d, J = 11.25 Hz, 2H), 4.19 (t, J = 24.00 Hz, 2H), 4.09 (d, J = 5.34 Hz, 2H), 1.84 (s, 2H). ^{13}C NMR (DMSO-d_6, 300 MHz, Supplementary Figure 9b): 166.47, 165.25, 156.18, 143.21 136.84, 135.99, 135.69, 135.27, 135.04, 134.89, 133.66, 129.56, 129.16, 128.92, 128.50, 128.15, 127.76, 125.78, 65.76, 64.78, 63.19, 42.43, 35.72, 17.91.

Synthesis of Tertiary Amine-Containing Monomer with Carbamate Side Linkage (DPA and DEA; Supplementary Figure 1c). Typical procedures employed for the synthesis of DPA monomer are as follows. *N,N*-Diisopropylaminoethanol (4.00 g, 27.5 mmol, 1.0 equiv.) and 2-isocyanatoethyl methacrylate (5.11 g, 33.0 mmol, 1.2 equiv.) were dissolved in anhydrous THF (50 mL), with the subsequent addition of catalytic amount of DBTL (50 μL). The reaction mixture was stirred at room temperature and monitored by TLC until the reaction was completed after ~6 h. The solvent was removed under reduced pressure, then the crude product was further purified by silica gel column chromatography, affording DPA monomer as a colorless liquid (7.18 g, yield: 87%). ^{1}H NMR ($CDCl_3$, δ, ppm, Supplementary Figure 10a): 6.12 (s, 1H), 5.59 (s, 1H), 4.93 (s, 1H), 4.22 (t, J = 10.62 Hz, 2H), 3.99 (t, J = 14.25 Hz, 2H), 3.50 (dd, J = 5.52, 5.52, 2H), 2.99 (m, 2H), 2.62 (t, J = 14.19, 2H), 1.95 (s, 3H), 1.02 (d, J = 13.38 Hz, 12H).

According to similar procedures, DEA monomer was also obtained as a colorless liquid (6.32 g, yield: 85%). ^{1}H NMR ($CDCl_3$, δ, ppm, Supplementary Figure 10b): 6.12 (s, 1H), 5.59 (s, 1H), 5.04 (s, 1H), 4.22 (t, J = 10.62 Hz, 2H), 4.14 (t, J = 15.96 Hz, 2H), 3.50 (dd, J = 5.43, 5.46, 2H), 2.99 (m, 2H), 2.68 (t, J = 11.88, 2H), 2.59 (dd, J = 7.17, 7.11, 4H), 1.95 (s, 3H), 1.03 (t, J = 14.28 Hz, 6H).

Synthesis of PEO_{45}-b-P($NCMA_x$-co-DPA_{1-x})$_n$ Diblock Copolymers and PEO_{45}-b-$PNCMA_m$-b-$PDPA_n$ Triblock Copolymers (Supplementary Figure 2). Typical procedures employed for the synthesis of PEO_{45}-*b*-P($NCMA_x$-*co*-DPA_{1-x})$_n$ diblock copolymer via RAFT polymerization are as follows. NCMA (1.50 g, 3.40 mmol, 20 equiv.), DPA (1.02 g, 3.40 mmol, 20 equiv.), PEO-CTA (0.39 g, 0.17 mmol, 1.0 equiv.), AIBN (3.4 mg, 0.021 mmol, 0.125 equiv.), and 1,4-dioxane (3 mL) were charged into a reaction tube equipped with a magnetic stirring bar. The solution mixture was degassed by three freeze-pump-thaw cycles and sealed under vacuum. The reaction tube was immersed into an oil bath thermostated at 70 ^{o}C and stirred for 7 h, and then quenched into liquid nitrogen and opened. The mixture was precipitated into an excess of diethyl ether. The above dissolution-precipitation cycle was repeated for three times and the target diblock copolymer was obtained as a yellowish powder (2.25 g, 74% yield). GPC analysis revealed an M_n of 12.7 kDa and an M_w/M_n of 1.26

(Supplementary Table 1, Supplementary Figure 11). The total degree of polymerization, DP, of P(NCMA-*co*-DPA) block was determined to be 29, with the NCMA molar fraction being ~0.55, by ^{1}H NMR analysis. Thus, the diblock copolymer was denoted as PEO_{45}-*b*-P($NCMA_{0.55}$-*co*-$DPA_{0.45}$)$_{29}$. According to similar procedures, PEO_{45}-*b*-$PNCMA_{17}$-*b*-$PDPA_{21}$ and PEO_{45}-*b*-P($NCMA_{0.49}$-*co*-$DEA_{0.51}$)$_{32}$ block copolymers were also synthesized and characterized by ^{1}H NMR and GPC (Supplementary Table 1; Supplementary Figures 12 and 13).

Synthesis of PEO_{45}-b-P($DCMA_x$-co-DPA_{1-x})$_n$ Amphiphilic Diblock Copolymer (Supplementary Figure 3). DCMA (1.50 g, 2.72 mmol, 20 equiv.), DPA (0.82 g, 2.72 mmol, 20 equiv.), PEO-CTA (0.32 g, 0.14 mmol, 1.0 equiv.), AIBN (3.0 mg, 0.018 mmol, 0.125 equiv.), and 1,4-dioxane (3 mL) were charged into a reaction tube equipped with a magnetic stirring bar. The mixture was degassed by three freeze-pump-thaw cycles and then sealed under vacuum. The reaction tube was immersed into an oil bath thermostated at 70 ^oC and stirred for 8 h, and quenched into liquid nitrogen and opened. The mixture was then precipitated into an excess of diethyl ether. The above dissolution-precipitation cycle was repeated for three times, affording the target diblock copolymer as a yellowish powder (2.06 g, 78% yield). GPC analysis revealed an M_n of 13.3 kDa and an M_w/M_n of 1.22 (Supplementary Table 1). The total DP of P(DCMA-*co*-DPA) block was determined to be 33, with the NCMA molar fraction being ~0.45, by ^{1}H NMR analysis (Supplementary Figure 14). Thus, the diblock copolymer was denoted as PEO_{45}-*b*-P($DCMA_{0.45}$-*co*-$DPA_{0.55}$)$_{33}$.

Synthesis of Dye-Functionalized Amphiphilic Block Copolymers (Supplementary Figure 4). Schematics of the synthesis of dye-functionalized amphiphilic diblock copolymers, PEO_{45}-*b*-P($NCMA_x$-*co*-DPA_{1-x})$_n$-*Nile red* and PEO_{45}-*b*-P($NCMA_x$-*co*-DPA_{1-x})$_n$-*naphthalimide*, are shown in Supplementary Figure 5. Typical procedures employed for the synthesis of PEO_{45}-*b*-P($NCMA_{0.55}$-*co*-$DPA_{0.45}$)$_{29}$-*Nile red* are as follows. PEO_{45}-*b*-P($NCMA_{0.55}$-*co*-$DPA_{0.45}$)$_{29}$ (500 mg, 0.038 mmol, 1.0 equiv. terminal trithiocarbonate moieties) was dissolved in methanol (5 mL) and cooled to 0 ^oC in an ice-water bath; under magnetic stirring, $NaBH_4$ (14 mg, 0.38 mmol, 10 equiv.) was added. After the color of reaction mixture changed from yellowish to colorless, 10 mL dry toluene was added. The mixture was azeotropically

evaporated to dryness under reduced pressure and precipitated into an excess of diethyl ether. The dissolution-precipitation cycle was repeated for three times, affording thiol-functionalized PEO_{45}-*b*-P($NCMA_{0.55}$-*co*-$DPA_{0.45}$)$_{29}$-*SH* as a white powder (452 mg, 90% yield).

Next, P($NCMA_{0.55}$-*co*-$DPA_{0.45}$)$_{29}$-*SH* (400 mg, 0.031 mmol, 1.0 equiv.) and Nile red-MA monomer (85 mg, 0.16 mmol, 5.0 equiv.) were dissolved in THF (5 mL). Triethylamine (31 mg, 0.31 mmol, 10 equiv.) was added and the reaction mixture was stirred for 5 h at room temperature. The mixture was concentrated under reduced pressure and then precipitated into an excess of diethyl ether for three times, affording PEO_{45}-*b*-P($NCMA_{0.55}$-*co*-$DPA_{0.45}$)$_{29}$-*Nile red* as a red solid (371 mg, yield: 89%).

PEO_{45}-*b*-P($NCMA_{0.55}$-*co*-$DPA_{0.45}$)$_{29}$-*naphthalimide* was synthesized as follows (Supplementary Figure 5b). For the synthesis of naphthalimide-containing methacrylate monomer (naphthalimide-MA), **5** was synthesized at first according to literature procedures.[4] Next, 2-isocyanatoethyl methacrylate (0.16 g, 1.06 mmol, 1.2 equiv.) and **5** (0.30 g, 0.884 mmol, 1.0 equiv.) were dissolved in anhydrous THF (15 mL), with the subsequent addition of catalytic amount of DBTL (20 μL). The reaction mixture was stirred at room temperature and monitored by TLC until the reaction was completed after ~8 h. The solvent was removed under reduced pressure, the crude product was further purified by silica gel column chromatography, affording naphthalimide-MA monomer as a yellowish solid (0.37 g, yield: 86%). ^{1}H NMR ($CDCl_3$, δ, ppm): 8.58 (d, 1H), 8.52 (d, 1H), 8.42 (d, 1H), 7.69 (t, 1H), 7.22 (d, 1H), 6.09 (s, 1H), 5.56 (s, 1H), 4.96 (s, 1H), 4.44 (dd, 4H), 4.16 (t, 2H), 3.45 (dd, 2H), 3.32 (t, 4H), 2.77 (br., 4H), 2.46 (s, 3H), 1.91 (s, 3H).

Finally, naphthalimide-functionalized PEO_{45}-*b*-P($NCMA_x$-*co*-DPA_y)-*naphthalimide* was synthesized according to similar procedures employed for the synthesis of Nile red-labeled amphiphilic block copolymer, PEO_{45}-*b*-P($NCMA_{0.55}$-*co*-$DPA_{0.45}$)$_{29}$-*Nile red*. It was obtained as a light green solid (328 mg, 90%). In addition, two types of control block copolymers without tertiary amine comonomers, PEO_{45}-*b*-$PNCMA_{30}$ and PEO_{45}-*b*-PPA_{26}, were also synthesized according to similar procedures (Supplementary Figures 5 and 10).

Characterization

^1H NMR (300 MHz) and ^{13}C NMR (75 MHz) spectra were acquired on a 300 MHz Bruker instrument. Molecular weight and molecular weight distributions, M_w/M_n, were determined by gel permeation chromatography (GPC) equipped with a Waters 1515 pump and a Waters 2414 differential refractive index detector (set at 30 ℃). It used a series of three linear Styragel columns at an oven temperature of 45 ℃. The eluent was THF at a flow rate of 1.0 mL/min. A series of low polydispersity polystyrene standards were employed for calibration. The degrees of polymerization, DPs, were determined by ^1H NMR analysis. UV/vis absorbance and optical transmittance experiments were conducted on a TU-1810 double-beam UV-Vis spectrophotometer (Beijing Puxi General Instrumental Co., Ltd.). Fluorescence measurements were conducted on RF-5301/PC (Shimadzu) spectrofluorometer. Handheld UV lamp (365 nm, 8 mW/cm^2) was used for all experiments relevant to UV light irradiation. Dynamic laser light scattering (DLS) measurements were conducted on a Malvern Zetasizer Nano ZS instrument. Transmission electron microscope (TEM) measurements were conducted on a JEOL 2010 electron microscope. The samples for TEM observations were prepared by dropping 10 μL of aqueous dispersion of self-assembled aggregates onto copper grids successively coated with thin films of Formvar and carbon.

Supplementary Table 1 Structural parameters of amphiphilic diblock and triblock copolymers, and their corresponding self-assembled nanostructures in aqueous media.

Block Copolymers	M_n [a] /kDa	M_n [b] /kDa	M_w/M_n [b]	Aggregates Morphology [c]	<D_h> of polymersomes [d] /nm (μ_2/Γ^2)	<D_h> of PICsomes [e] /nm (μ_2/Γ^2)
**PEO_{45}-*b*-P($NCMA_{0.55}$-*co*-$DPA_{0.45}$)$_{29}$**	13.1	12.7	1.26	Vesicles	550 (0.10)	570 (0.09)
**PEO_{45}-*b*-$PNCMA_{17}$-*b*-$PDPA_{21}$**	16.0	15.5	1.31	Vesicles	530 (0.11)	770 (0.10)
**PEO_{45}-*b*-P($NCMA_{0.49}$-*co*-$DEA_{0.51}$)$_{32}$**	13.6	13.2	1.34	Vesicles	545 (0.08)	580 (0.09)
**PEO_{45}-*b*-P($DCMA_{0.45}$-*co*-$DPA_{0.55}$)$_{33}$**	13.7	13.3	1.22	Vesicles	640 (0.09)	675 (0.11)

[a] Determined by ^{1}H NMR analysis. [b] Obtained from GPC analysis using THF as eluent at a flow rate of 1.0 mL/min. [c] Self-assembly was conducted in aqueous media at 25 ^{o}C using the cosolvent approach. [d] Polymersome dispersion in aqueous media without external stimuli. [e] External stimuli-triggered formation of PICsomes in aqueous media. [d,e] Determined by dynamic light scattering (DLS) analysis.

Supplementary Table 2 Drug loading efficiency, loading content, and loaded drug concentration determined for drugs and model drugs encapsulated in PEO_{45}-*b*-P($NCMA_{0.55}$-*co*-$DPA_{0.45}$)$_{29}$ polymersomes, PEO_{45}-*b*-$PNCMA_{17}$-*b*-$PDPA_{21}$ polymersomes, and PEO_{45}-*b*-P($DCMA_{0.45}$-*co*-$PDPA_{0.55}$)$_{33}$ polymersomes, respectively.

Vesicles / Drug	PEO_{45}-*b*-P($NCMA_{0.55}$-*co*-$DPA_{0.45}$)$_{29}$ Polymersomes					PEO_{45}-*b*-$PNCMA_{17}$-*b*-$PDPA_{21}$ Polymersomes			PEO_{45}-*b*-P($DCMA_{0.45}$-*co*-$PDPA_{0.55}$)$_{33}$ Polymersomes	
Drug & Model drug	Gemcitabine	Dox·HCl	5-Fu	Coumarin 343	Calcein	Gemcitabine	Dox·HCl	Calcein	5-Fu	Dox·HCl
Loading Efficiency [a]	8.6%	8.6%	8.7%	8.9%	8.2%	8.5%	8.9%	8.4%	8.5%	8.8%
Drug Concentration (mM) [a]	1.07 [b]	1.07 [c]	1.09 [b]	1.11 [c]	1.02 [c]	1.06 [b]	1.11 [c]	1.05 [c]	1.06 [b]	1.10 [c]
Loading Content $wt_{drug}/(wt_{drug} + wt_{vesicles})$	61.5%	73.6%	41.5%	61.2%	76.0%	61.4%	76.3%	76.6%	40.8%	76.1%

[a] Determined at a polymersome concentration of 0.2 g/L. [b] Determined by UV-Vis absorbance. [c] Determined by fluorescence measurements.

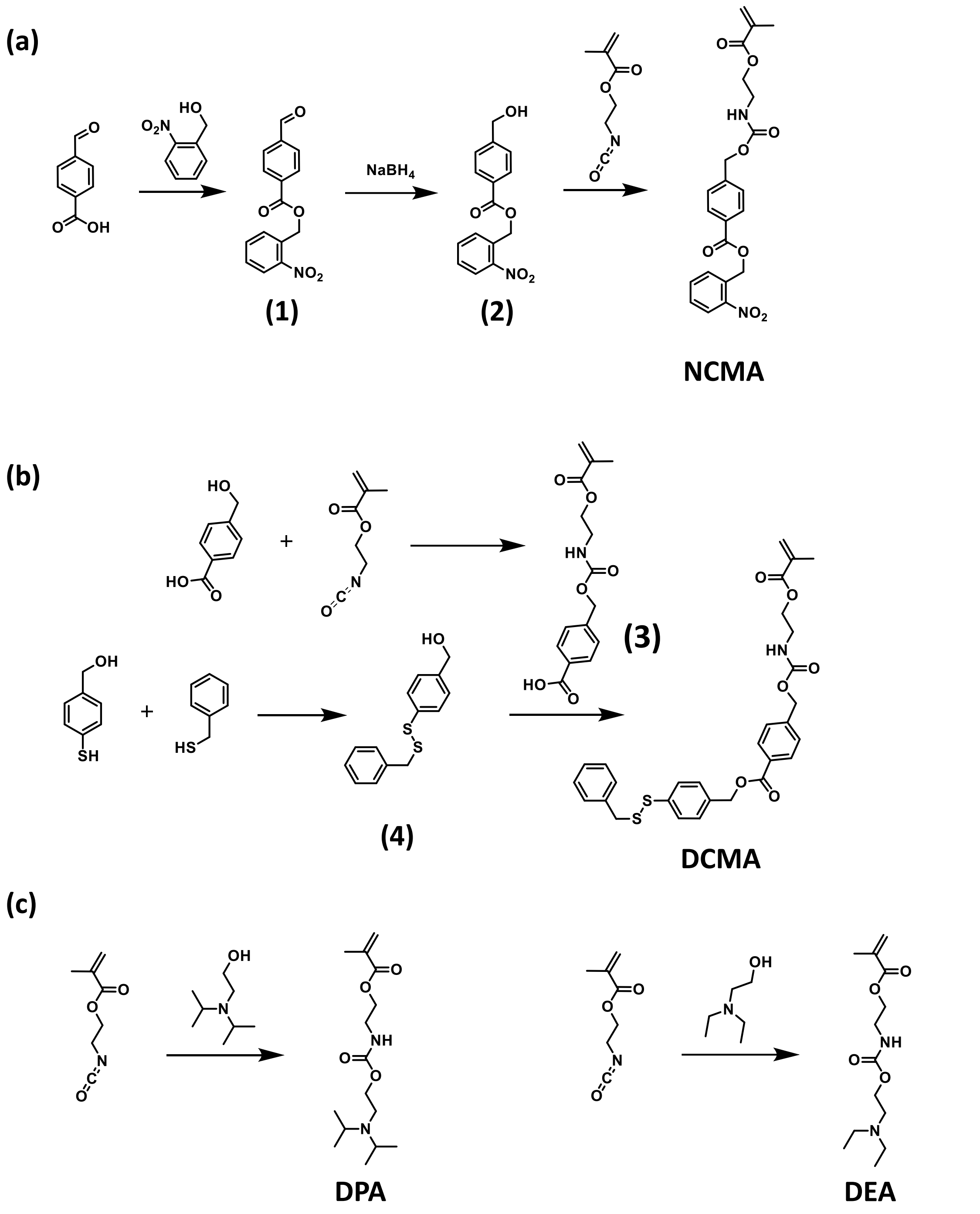

Supplementary Figure 1 Synthetic routes employed for the preparation of (a) 2-nitrobenzyl ester-photocaged carboxyl monomer (NCMA), (b) disulfide-caged carboxyl monomer (DCMA), and (c) two types of tertiary amine-containing monomers with carbamate linkages, DPA and DEA.

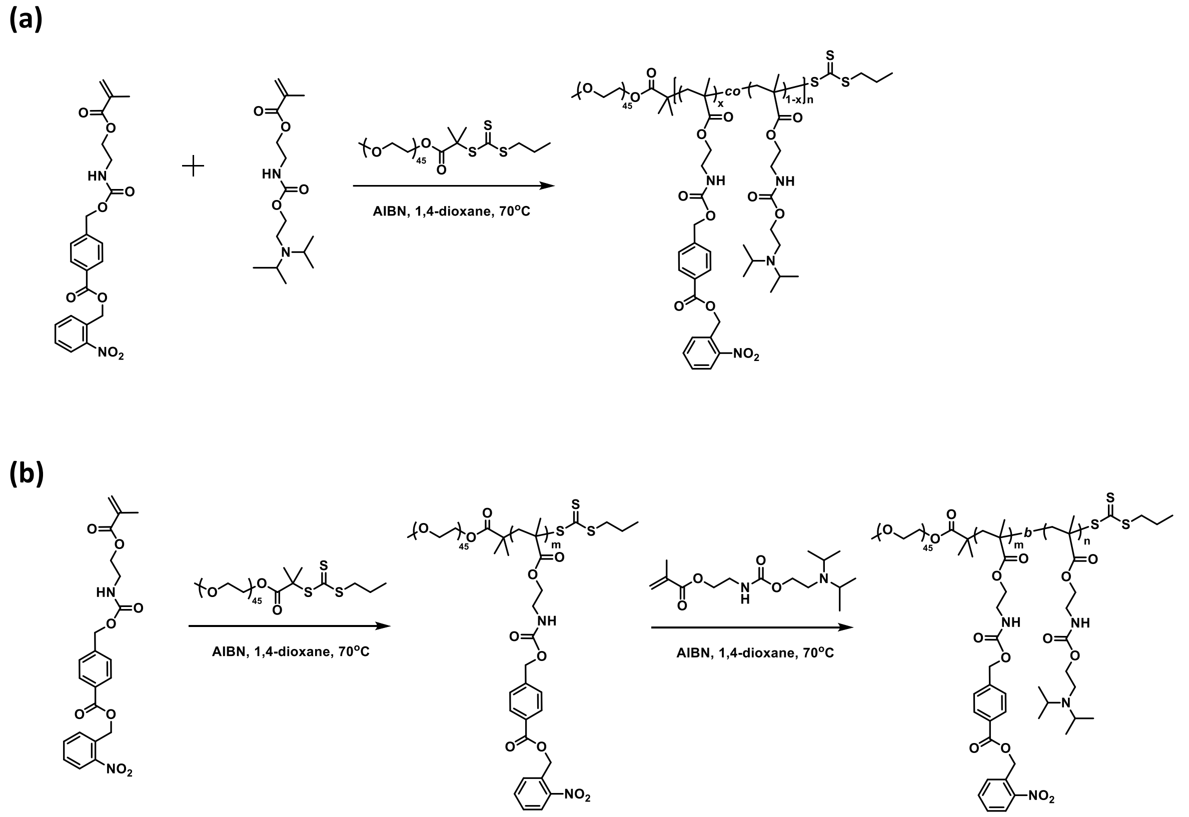

Supplementary Figure 2 Synthetic routes employed for the preparation of amphiphilic (a) PEO_{45}-*b*-P($NCMA_x$-*co*-DPA_{1-x})$_n$ diblock copolymers and (b) PEO_{45}-*b*-$PNCMA_m$-*b*-$PDPA_n$ triblock copolymers, respectively.

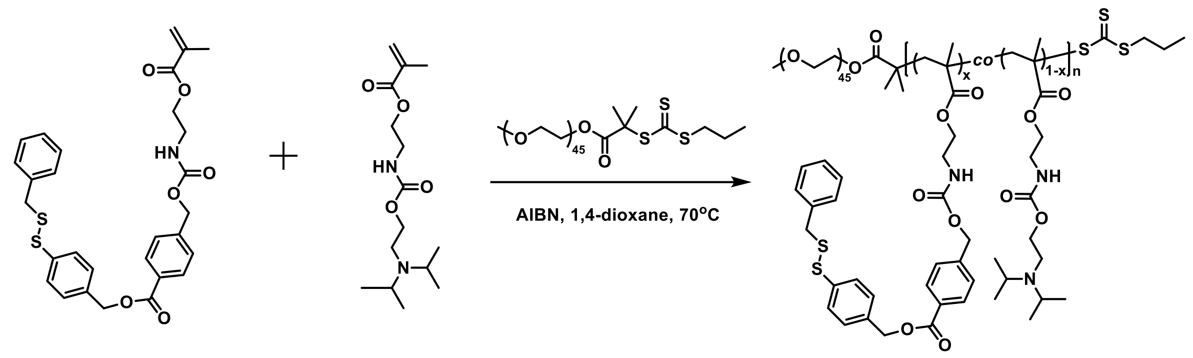

Supplementary Figure 3 Schematics of the preparation of amphiphilic PEO_{45}-*b*-P($DCMA_x$-*co*-DPA_{1-x})$_n$ diblock copolymers.

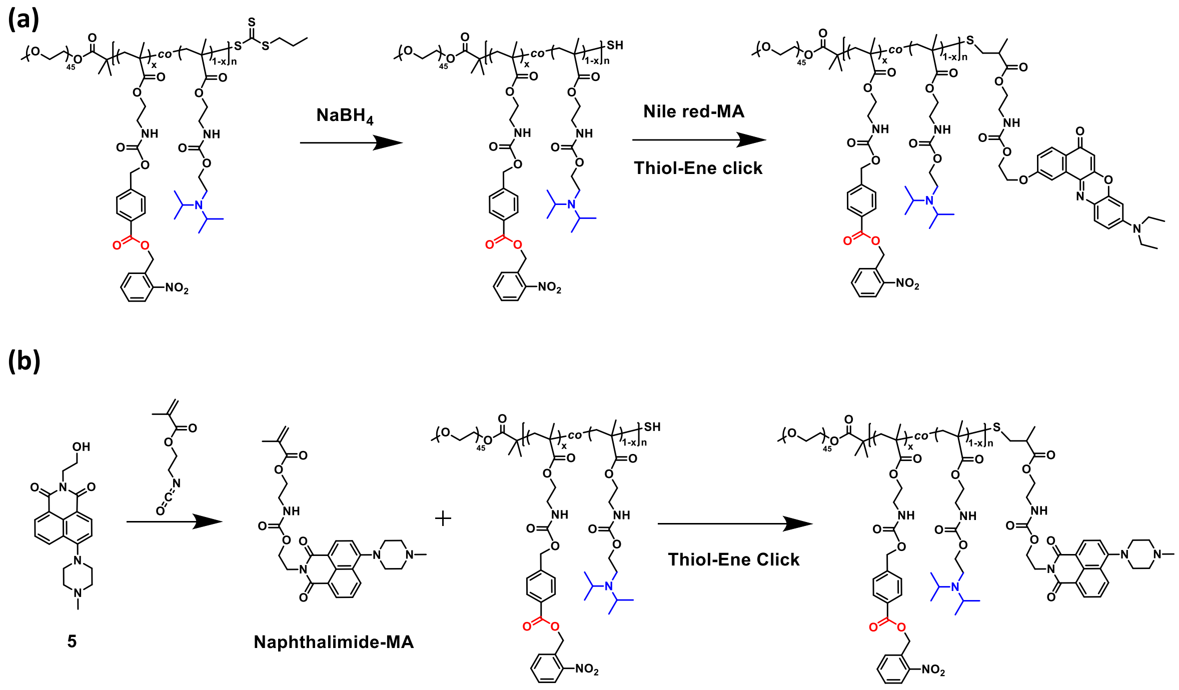

Supplementary Figure 4 Synthetic routes employed for the synthesis of (a) PEO_{45}-*b*-P($NCMA_x$-*co*-DPA_{1-x})$_n$-*Nile red* and (b) PEO_{45}-*b*-P($NCMA_x$-*co*-DPA_{1-x})$_n$-*naphthalimide*, respectively.

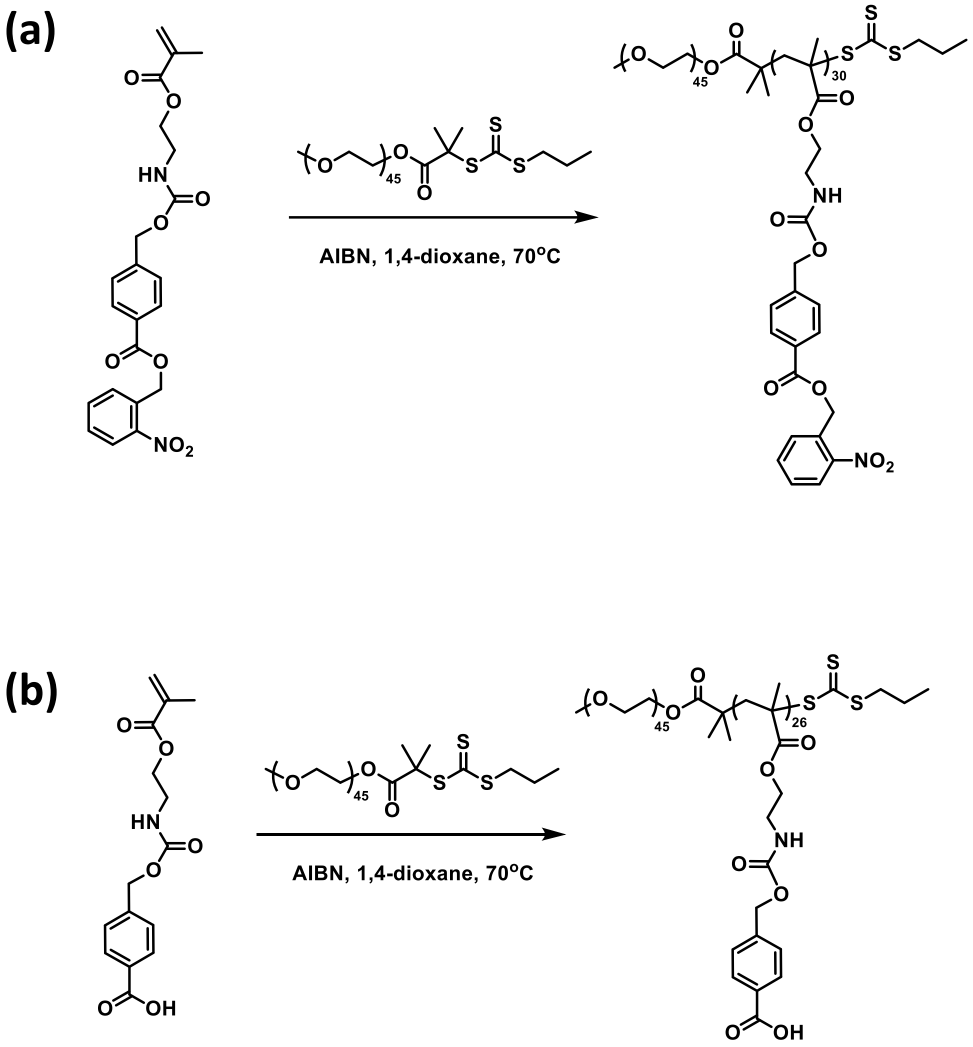

Supplementary Figure 5 Synthetic routes employed for the preparation of two types of amphiphilic diblock copolymers as control samples: (a) PEO_{45}-*b*-$PNCMA_{30}$ and (b) PEO_{45}-*b*-PPA_{26}.

(a)

(b)

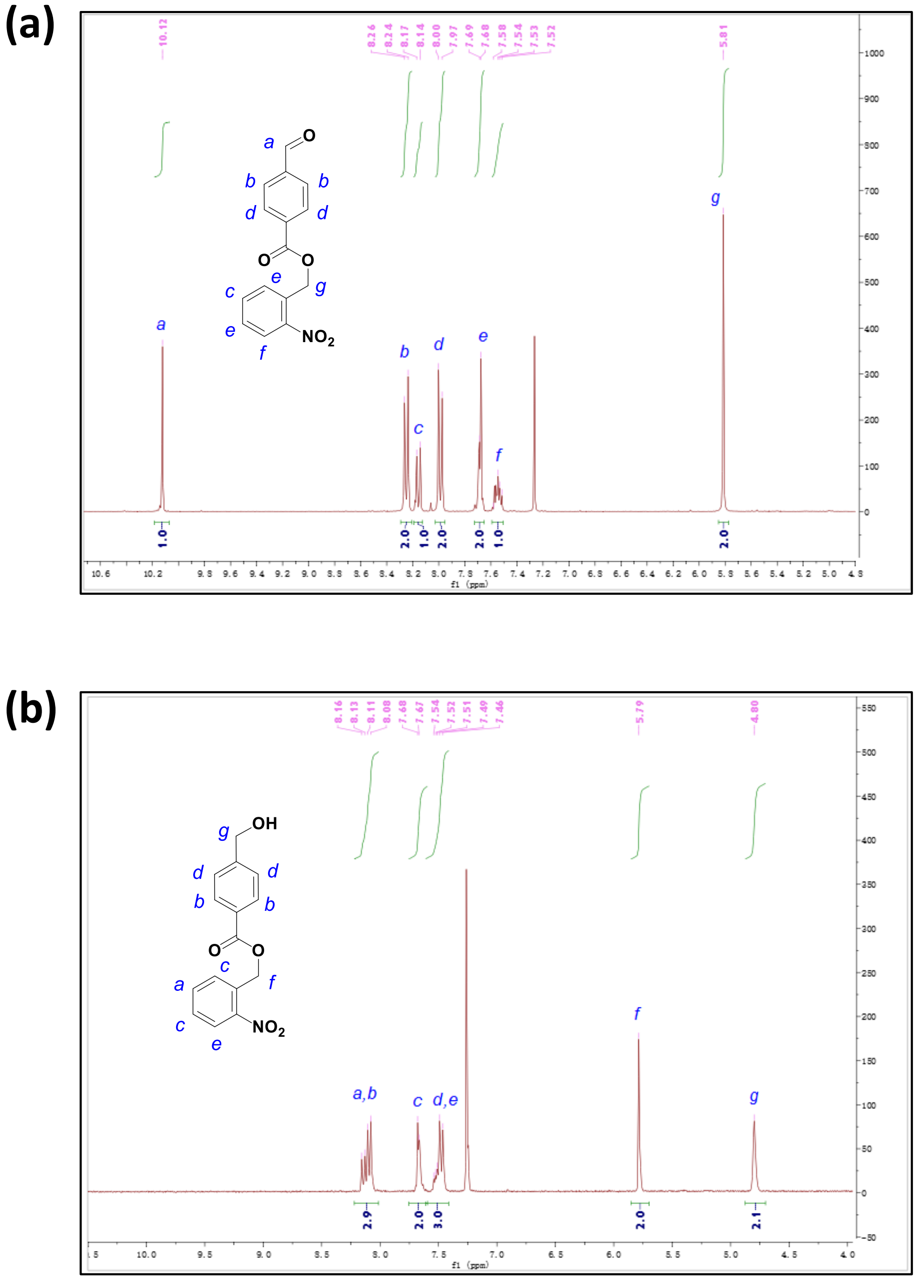

Supplementary Figure 6 ^{1}H NMR spectra recorded for (a) **1** and (b) **2** in $CDCl_3$.

(a)

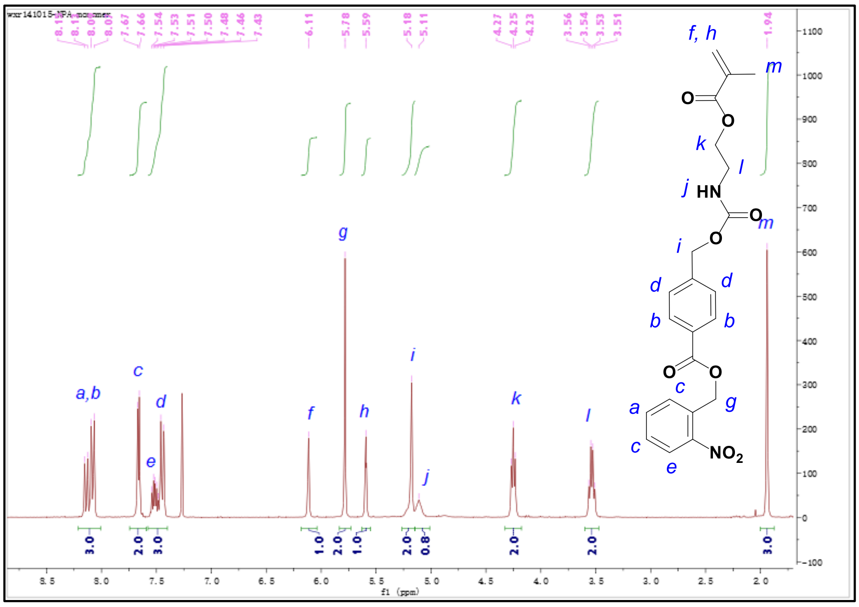

(b)

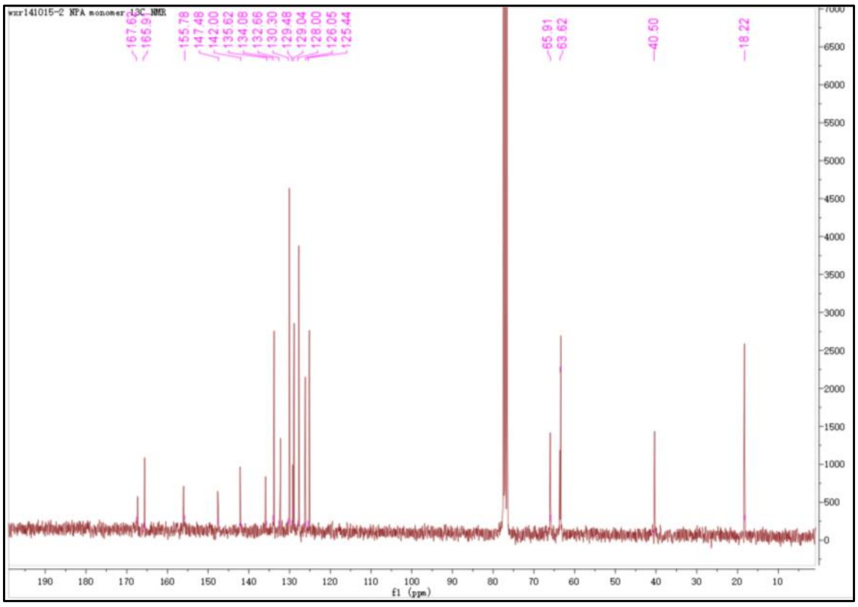

Supplementary Figure 7 (a) ^{1}H NMR and (b) ^{13}C NMR spectra recorded for NCMA monomer in $CDCl_3$.

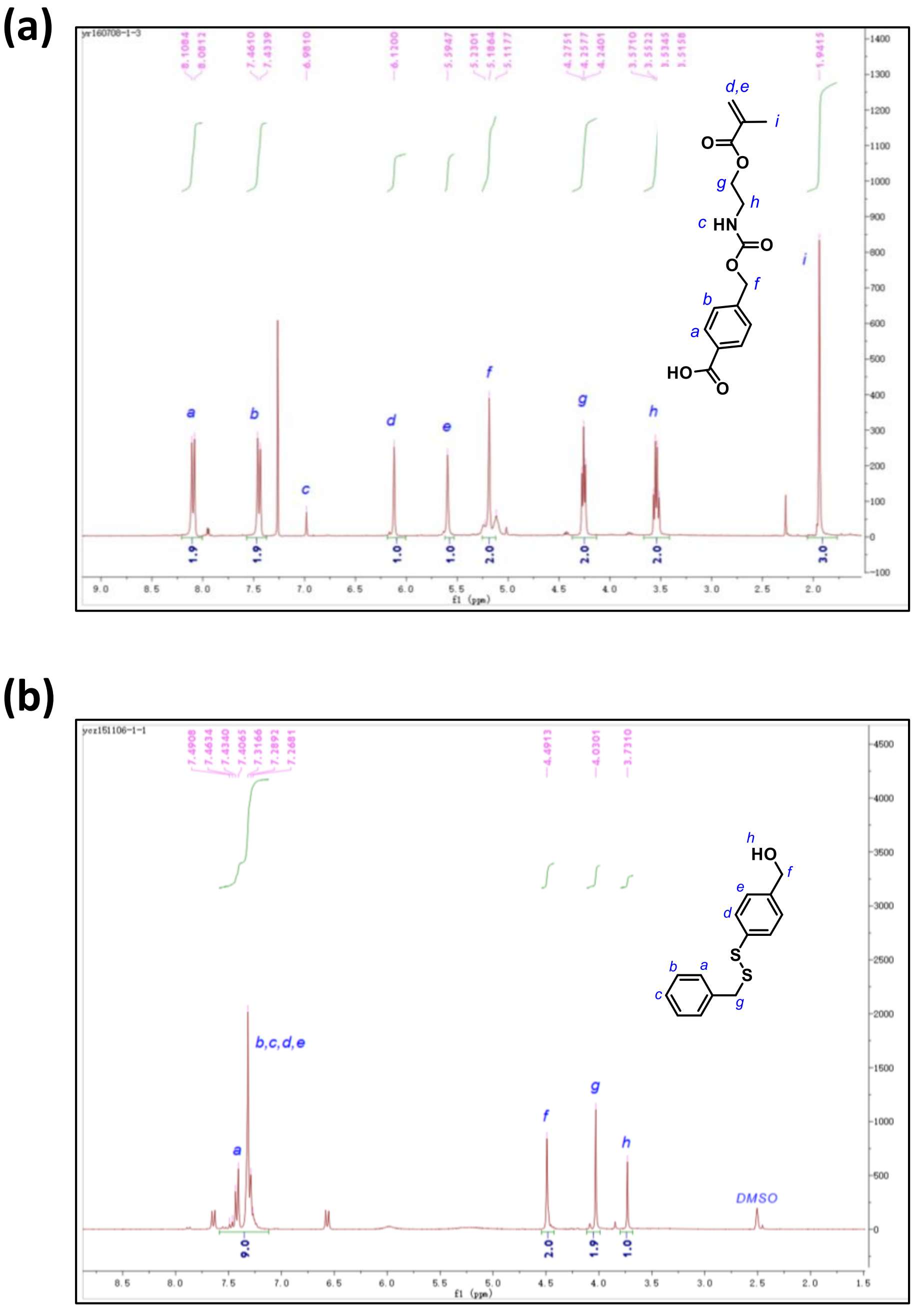

Supplementary Figure 8 ^{1}H NMR spectra recorded for (a) **3** in $CDCl_3$ and (b) **4** in DMSO-d_6.

(a)

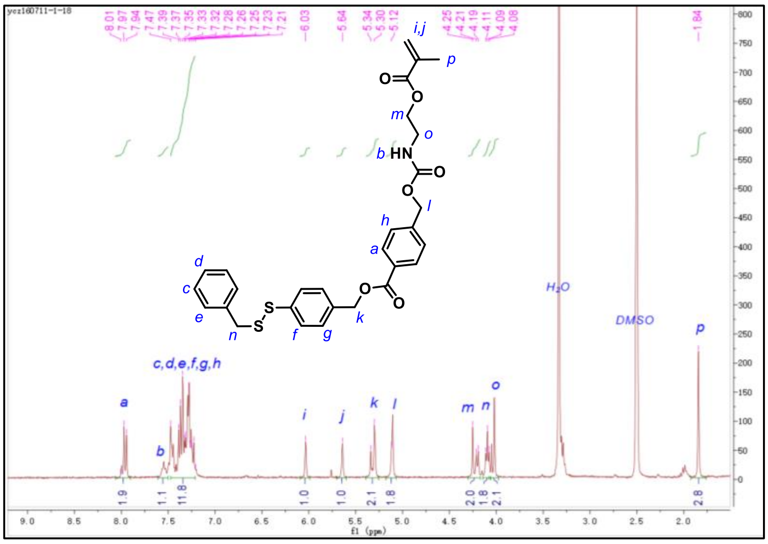

(b)

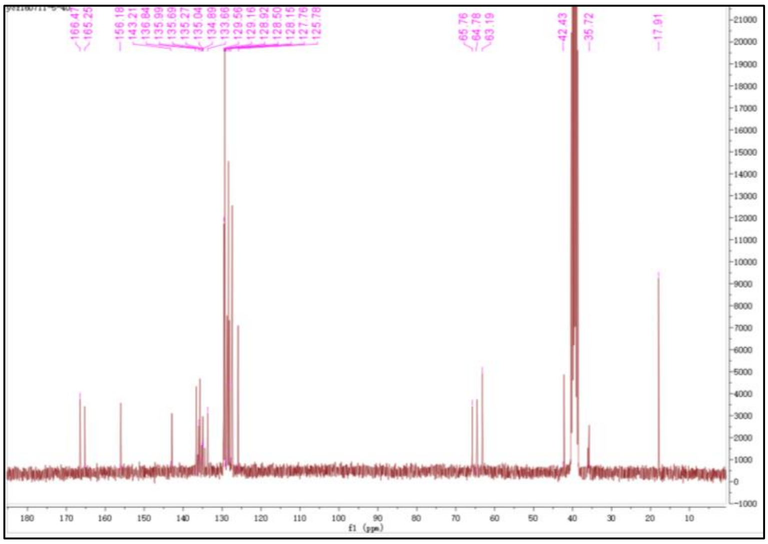

Supplementary Figure 9 (a) ^{1}H NMR and (b) ^{13}C NMR spectra recorded for DCMA monomer in DMSO-d_6.

(a)

(b)

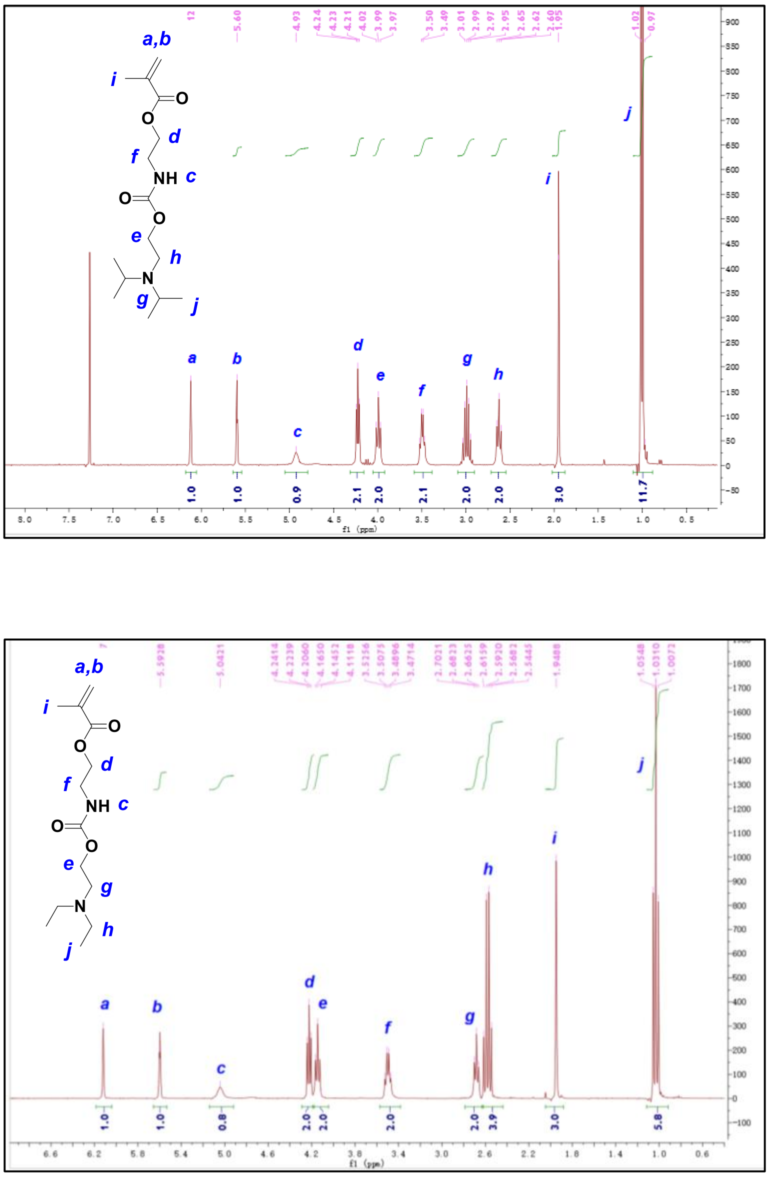

Supplementary Figure 10 ^{1}H NMR spectra recorded in $CDCl_3$ for two types of tertiary amine-containing monomers with carbamate linkages: (a) DPA and (b) DEA.

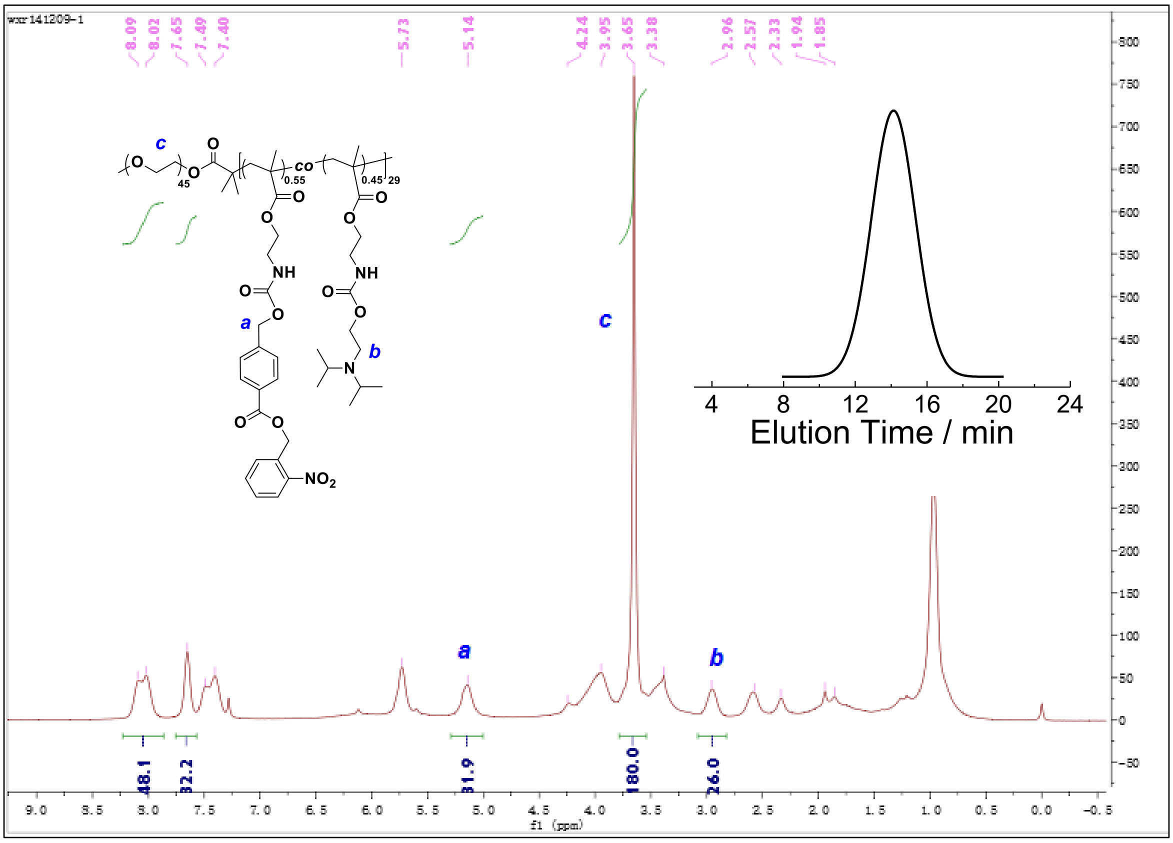

Supplementary Figure 11 ^{1}H NMR spectrum recorded for PEO$_{45}$-*b*-P(NCMA$_{0.55}$-*co*-DPA$_{0.45}$)$_{29}$ in $CDCl_3$. The inset shows GPC elution trace recorded for PEO$_{45}$-*b*-P(NCMA$_{0.55}$-*co*-DPA$_{0.45}$)$_{29}$ using THF as eluent.

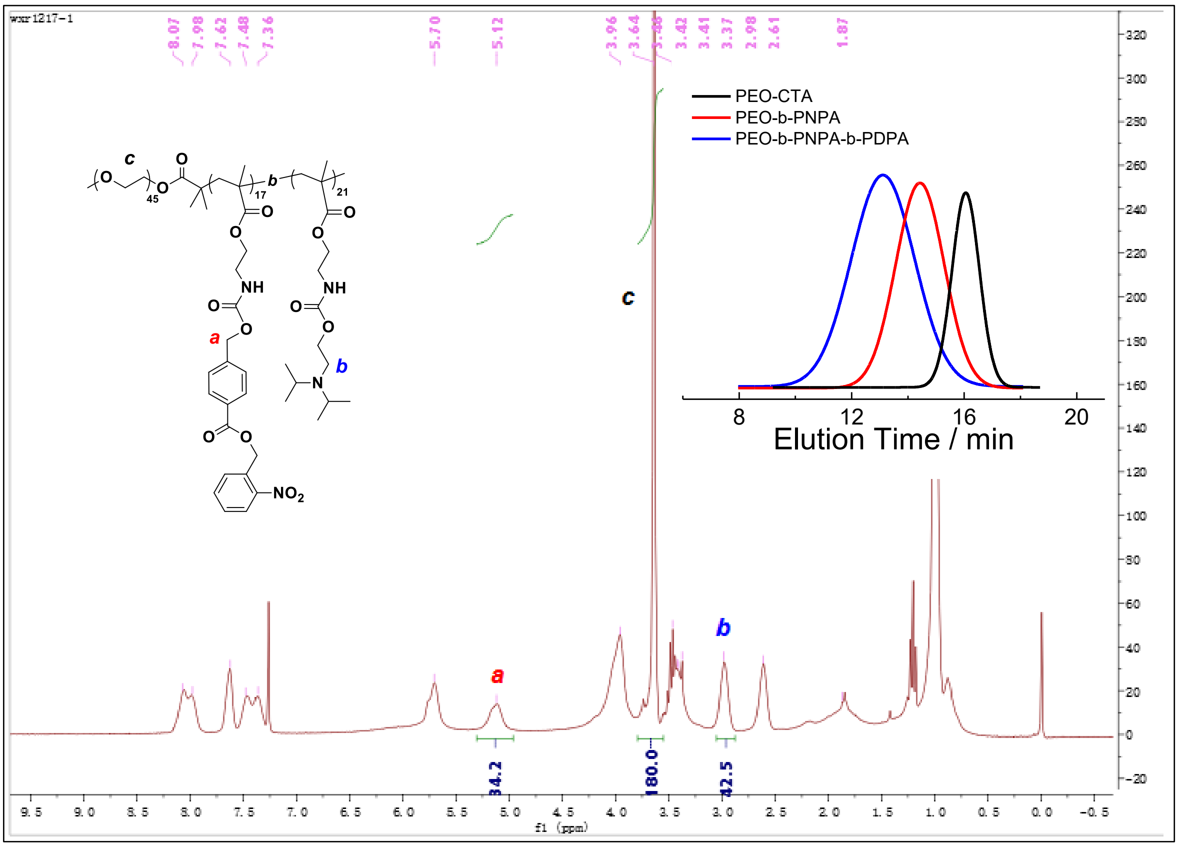

Supplementary Figure 12 ^{1}H NMR spectrum recorded for PEO$_{45}$-*b*-PNCMA$_{17}$-*b*-PDPA$_{21}$ in CDCl$_3$. The inset shows the GPC elution traces recorded for PEO-based macroRAFT agent, PEO$_{45}$-*b*-PNCMA$_{17}$, and PEO$_{45}$-*b*-PNCMA$_{17}$-*b*-PDPA$_{21}$ using THF as eluent.

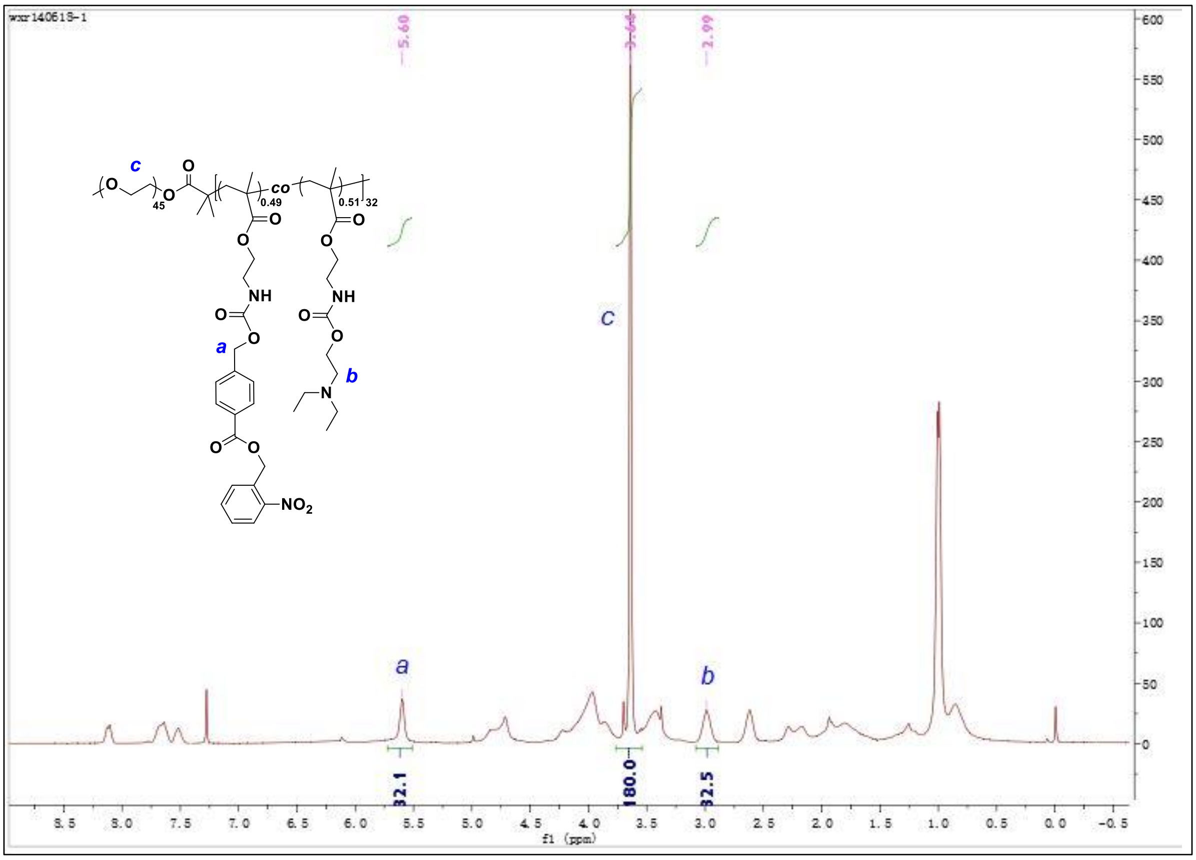

Supplementary Figure 13 ^{1}H NMR spectrum recorded for PEO_{45}-*b*-P($NCMA_{0.49}$-*co*-$DEA_{0.51}$)$_{32}$ in $CDCl_3$.

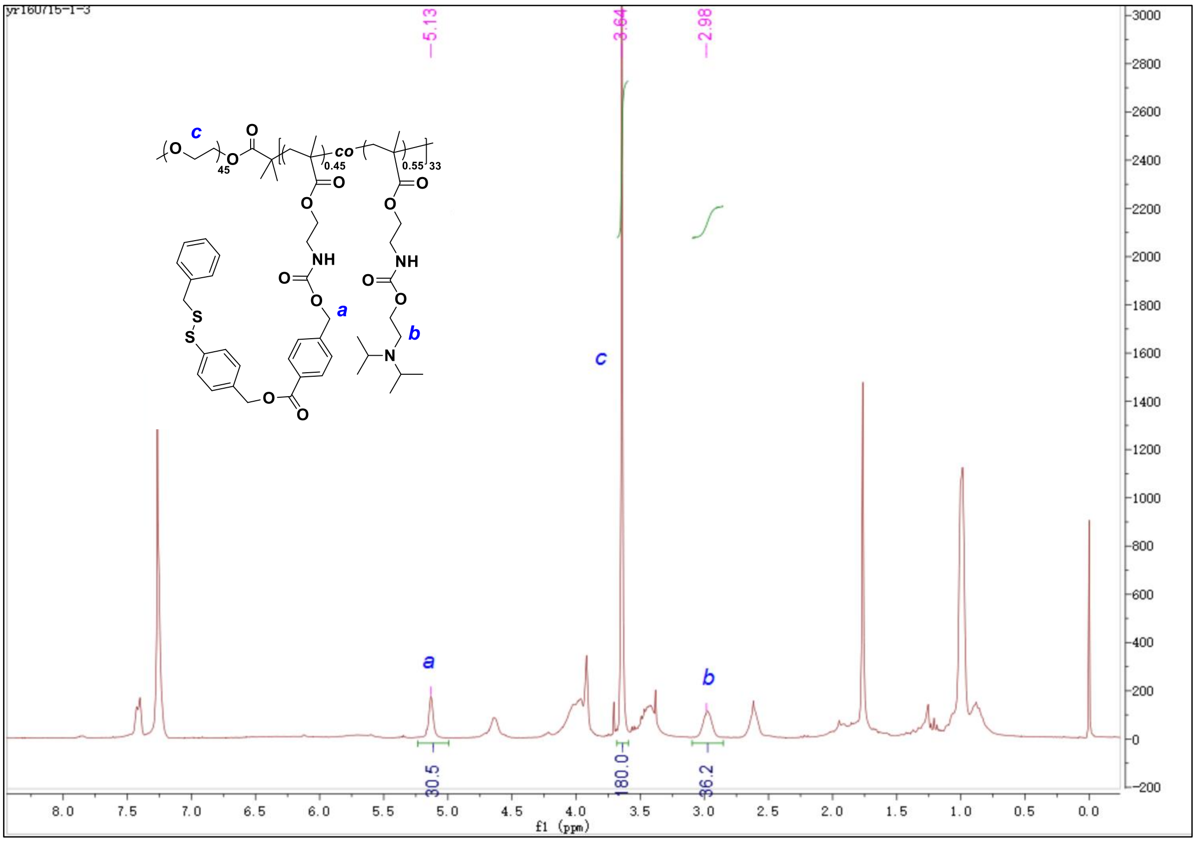

Supplementary Figure 14 ^{1}H NMR spectrum recorded for PEO$_{45}$-*b*-P(DCMA$_{0.45}$-*co*-DPA$_{0.55}$)$_{33}$ in $CDCl_3$.

(a)

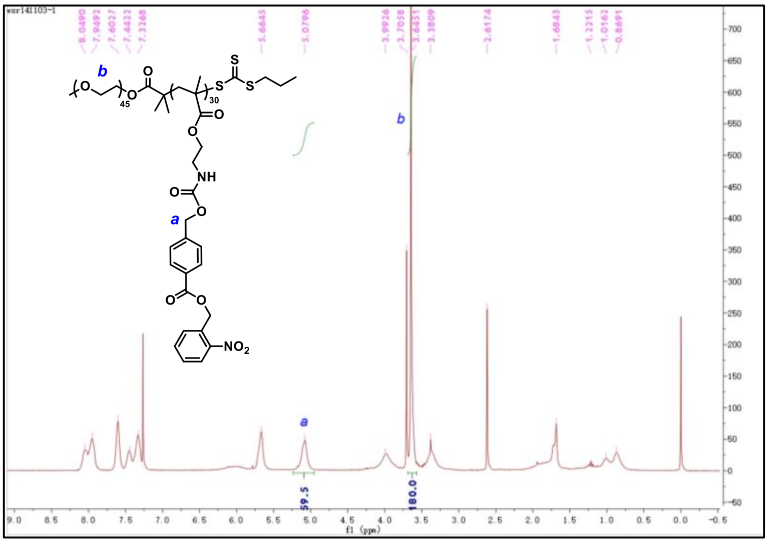

(b)

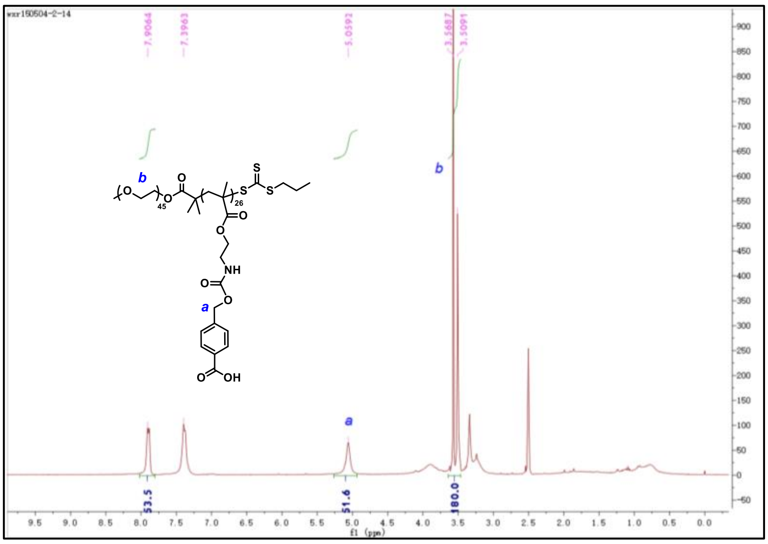

Supplementary Figure 15 ^{1}H NMR spectra recorded for two control BCPs: (a) PEO_{45}-*b*-$PNCMA_{30}$ in $CDCl_3$, and (b) PEO_{45}-*b*-PPA_{26} in DMSO-d_6, respectively.

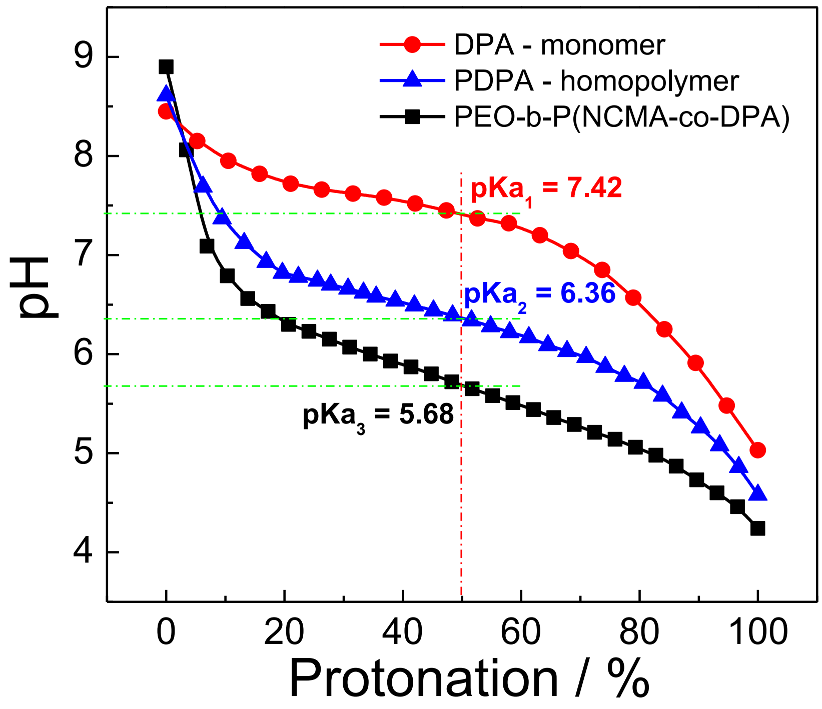

Supplementary Figure 16 pH-dependent evolution of degrees of protonation, *α*, determined for DPA monomer, PDPA homopolymer, and PEO_{45}-*b*-P($NCMA_{0.55}$-*co*-$DPA_{0.45}$)$_{29}$ diblock copolymer, respectively (25 ^{o}C, aqueous media).

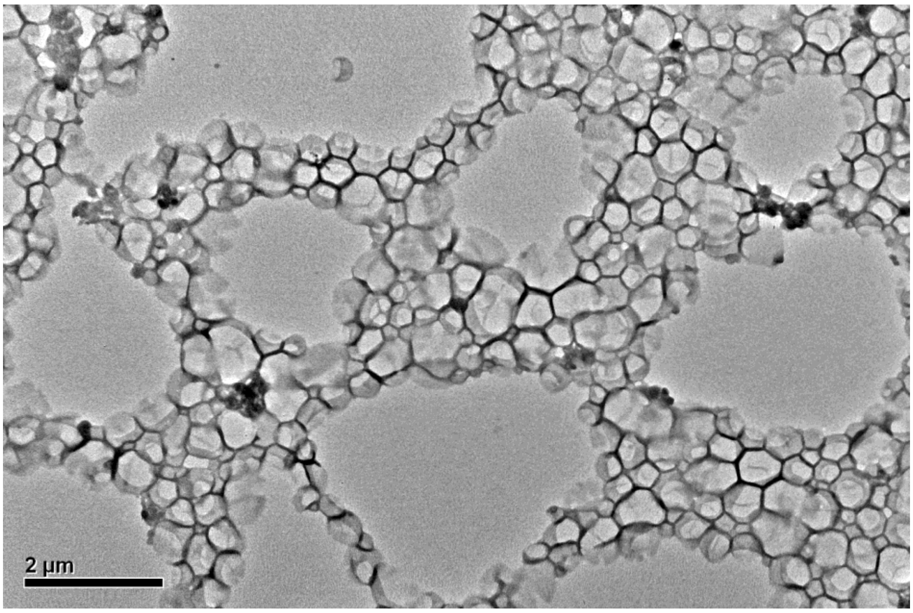

Supplementary Figure 17 TEM image recorded at an enlarged field view for PEO_{45}-*b*-$P(NCMA_{0.55}$-*co*-$DPA_{0.45})_{29}$ polymersomes.

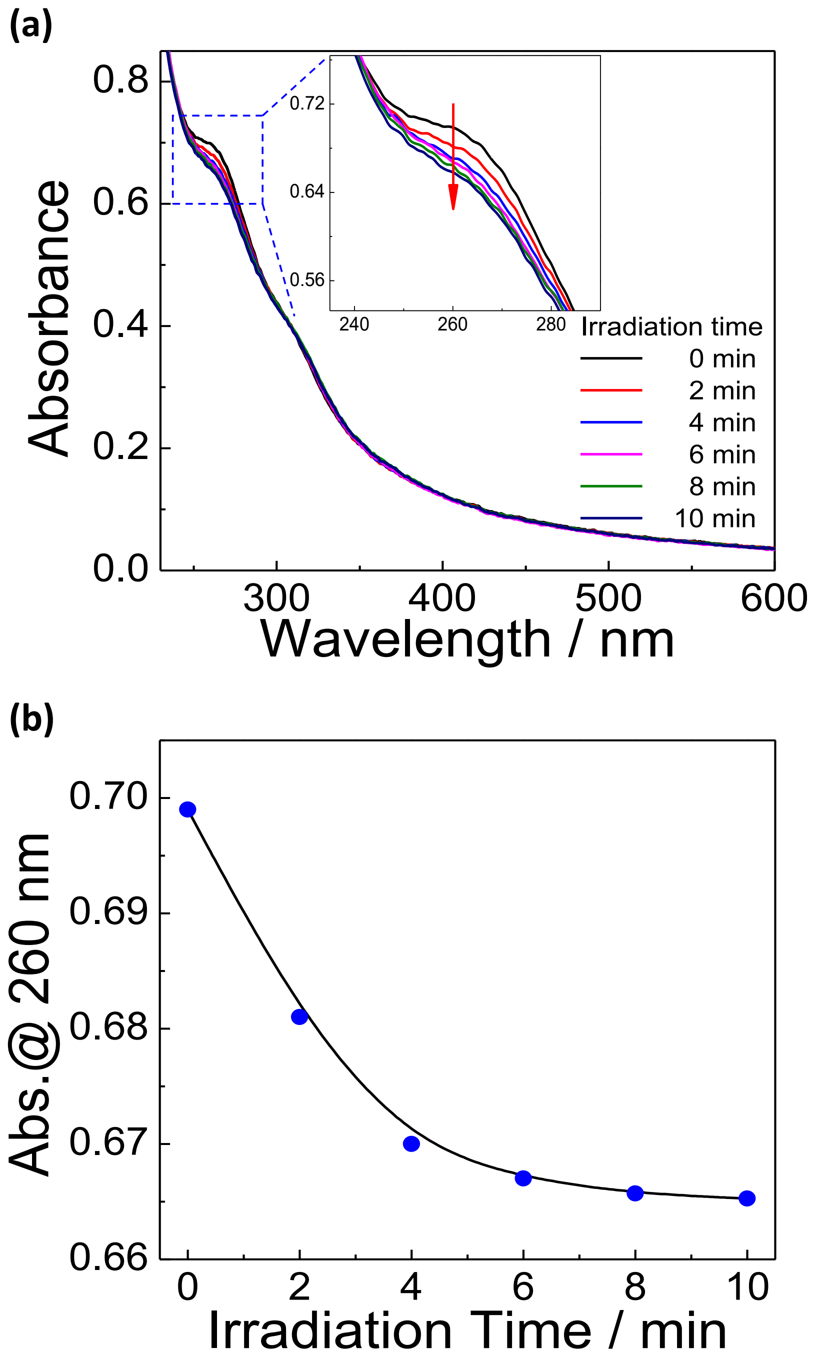

Supplementary Figure 18 (a) Irradiation time-dependence of UV/Vis absorbance spectra recorded for PEO_{45}-*b*-P($NCMA_{0.55}$-*co*-$DPA_{0.45}$)$_{29}$ vesicles in neutral aqueous media (vesicles, 0.1 g/L, 25 ^{o}C, UV 0-10 min, 365 nm, 8 mW/cm^2). The inset shows enlarged UV absorption spectra in the range of 235~290 nm. (b) The change of UV absorbance at λ_{abs} = 260 nm with UV irradiation time.

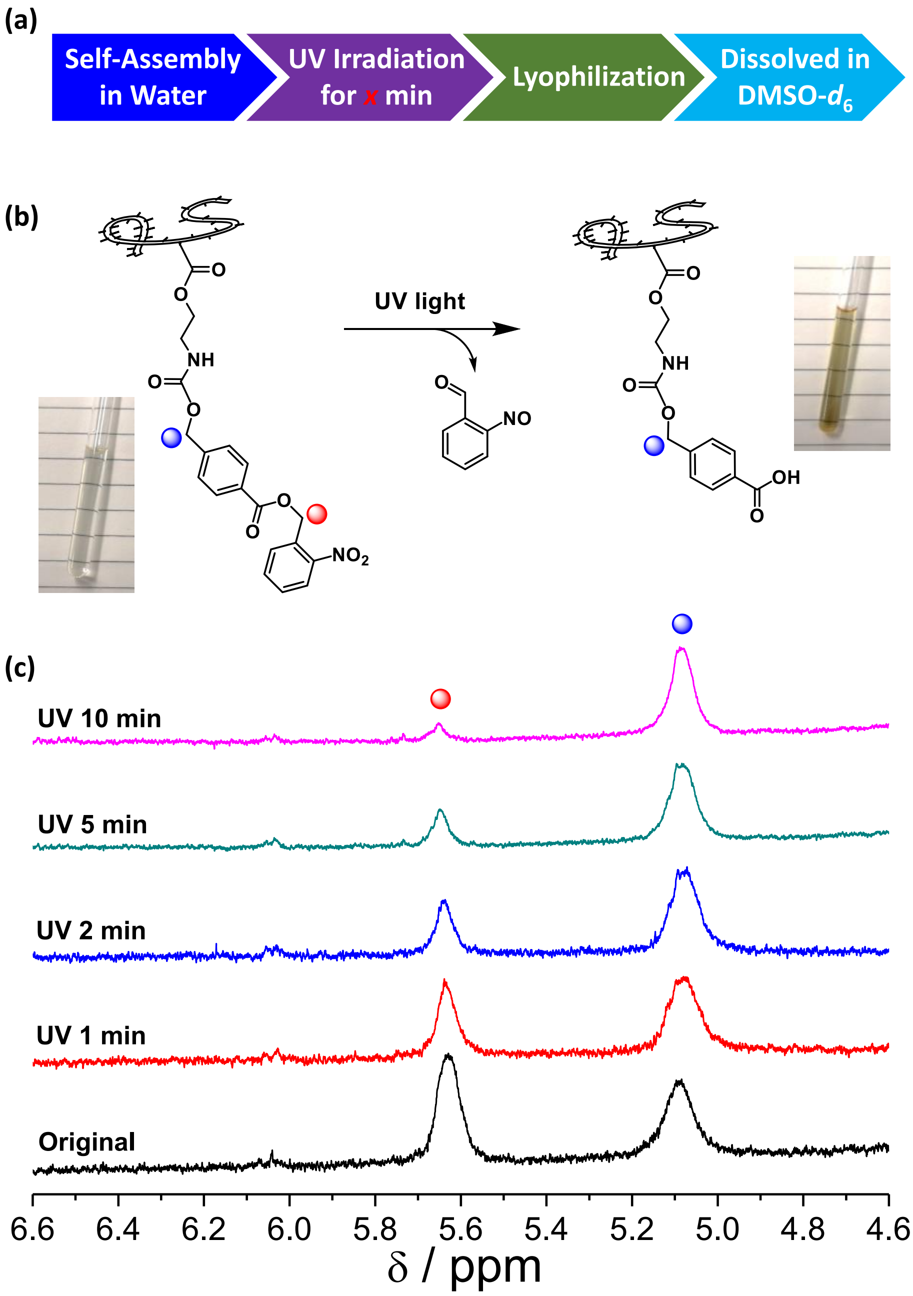

Supplementary Figure 19 ^{1}H NMR characterization of UV-triggered carboxyl decaging process for PEO_{45}-*b*-P($NCMA_{0.55}$-*co*-$DPA_{0.45}$)$_{29}$ polymersomes. (a) Brief illustration of experimental procedures. (b) Chemical structural changes of NCMA moieties upon UV irradiation. Inset images show DMSO-d_6 solutions of lyophilized powders obtained for polymersome dispersions before and after UV irradiation for 10 min; in both cases, lyophilized samples could be completely dissolved in DMSO-d_6. (c) UV irradiation duration time-dependent evolution of ^{1}H NMR spectra recorded in DMSO-d_6, demonstrating >98% photocleavage efficiency upon 10 min UV irradiation. All data were obtained at a polymersome concentration of 0.1 g/L; UV irradiation for 0-10 min, 365 nm, 8 mW/cm^2.

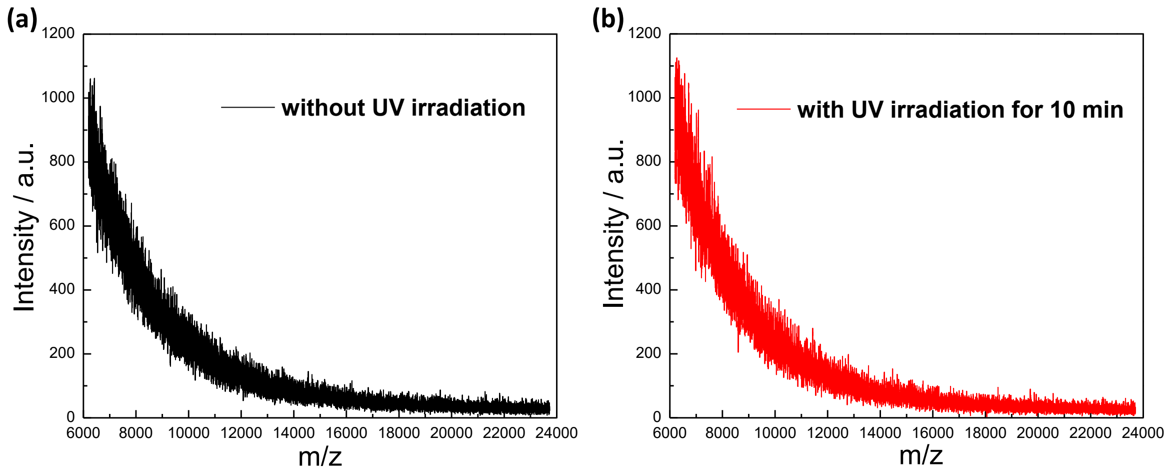

Supplementary Figure 20 MALDI-TOF MS data of lyophilized PEO_{45}-*b*-P($NCMA_{0.55}$-*co*-$DPA_{0.45}$)$_{29}$ polymersome dispersions (a) before and (b) after 10 min UV irradiation. Note that no reliable signals could be obtained in both cases, which might be due to the high molar mass of block copolymer and/or low extent of ionization under MALDI-TOF MS characterization conditions.

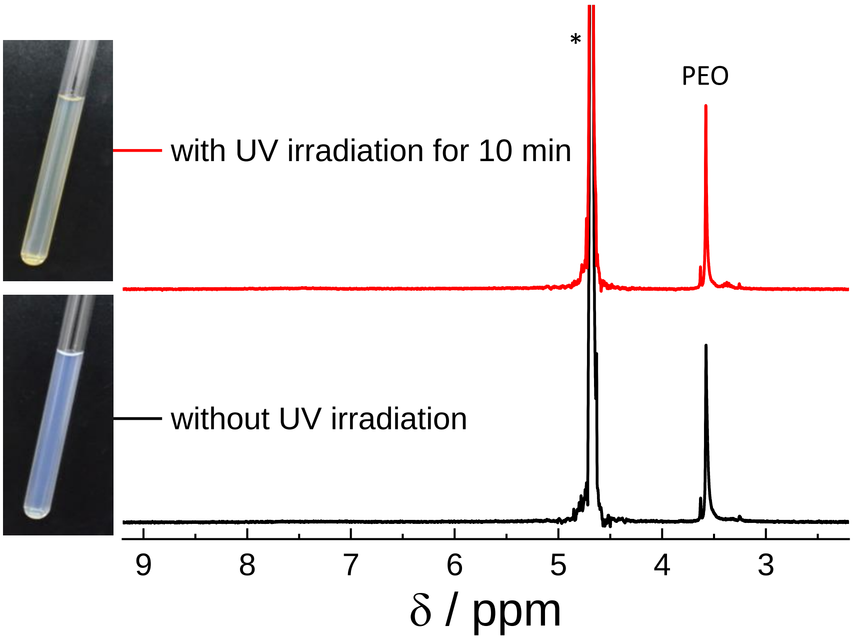

Supplementary Figure 21 ^{1}H NMR spectra recorded for PEO_{45}-*b*-P($NCMA_{0.55}$-*co*-$DPA_{0.45}$)$_{29}$ vesicle dispersions in D_2O (2.0 g/L) before and after UV irradiation, respectively. The insets show macroscopic images recorded for vesicular dispersions in NMR tube before (bottom) and after (top) UV irradiation (10 min, 365 nm, 8 mW/cm^2), respectively. The star (*) represents the solvent.

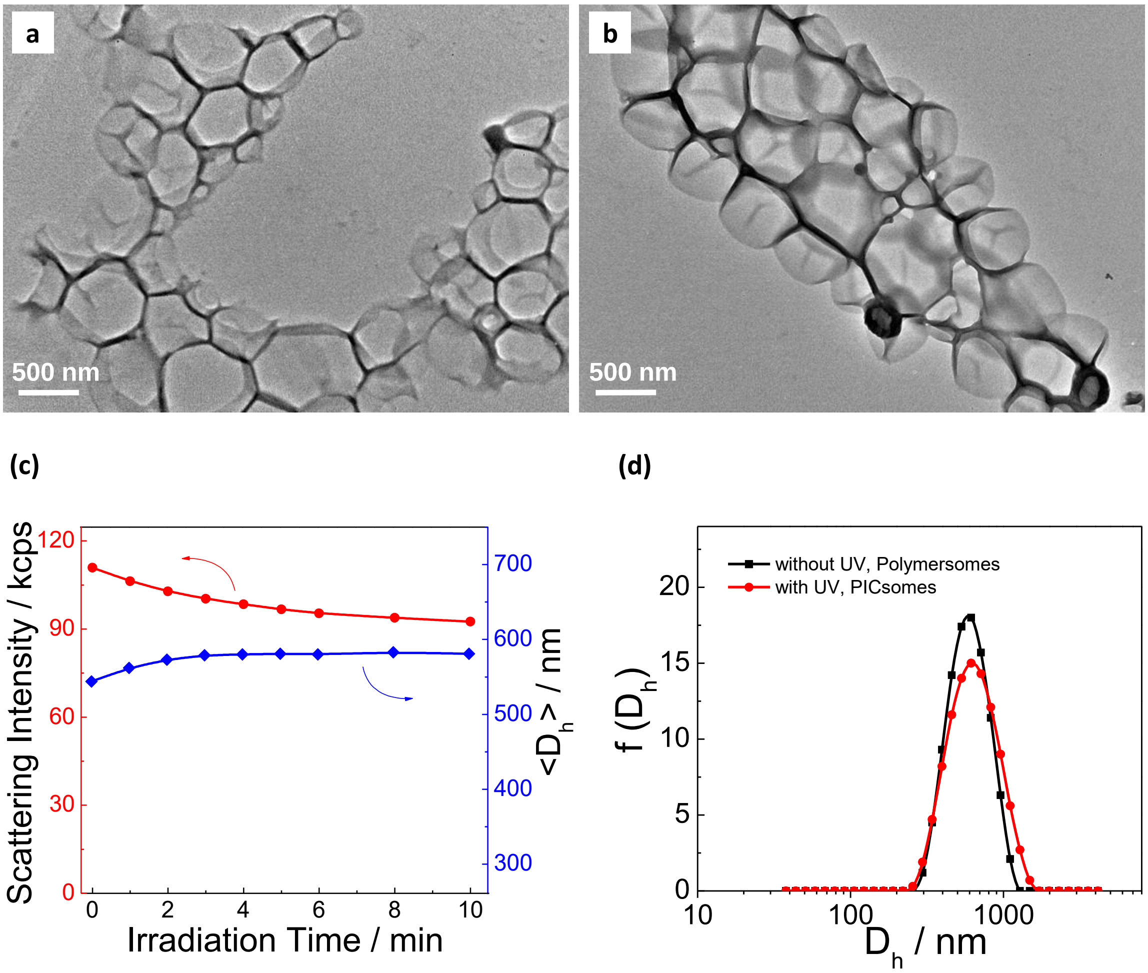

Supplementary Figure 22 (a,b) TEM images recorded for PEO$_{45}$-*b*-P(NCMA$_{0.49}$-*co*-PDEA$_{0.51}$)$_{32}$ polymersomes and corresponding PICsomes in neutral aqueous media, respectively. (c) Irradiation duration-dependent evolution of scattered light intensities and <D_h> of polymersomes and corresponding PICsomes upon UV irradiation in neutral aqueous media. (d) Intensity-average D_h distributions of vesicles before and after UV irradiation in aqueous media. All experiments were conducted at a polymer concentration of 0.1 g/L in phosphate buffer (pH 7.4, 10 mM).

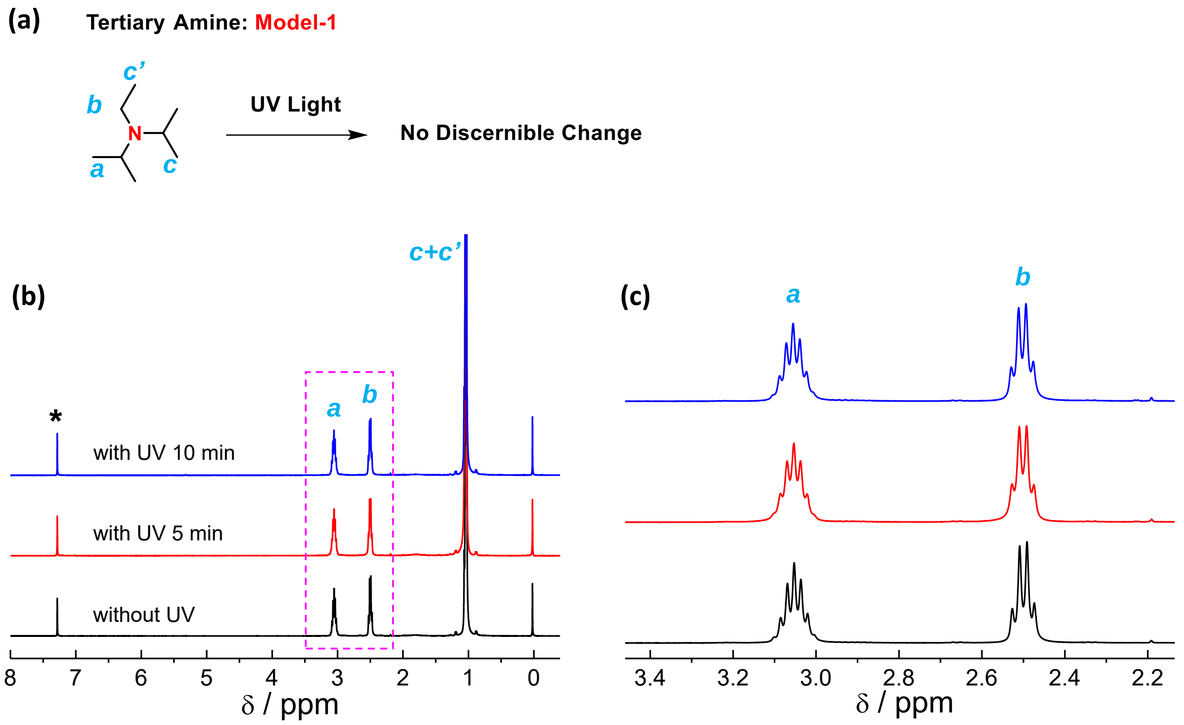

Supplementary Figure 23 (a) Schematics of photostability assay of *N*,*N*-diisopropylethylamine against UV irradiation. (b) ^1H NMR spectra recorded in $CDCl_3$ for *N*,*N*-diisopropylethylamine without and with UV irradiation for 5 min and 10 min, respectively (UV light, 365 nm, 8 mW/cm^2). (c) Enlarged ^1H NMR spectra in the range of 2.2~3.4 ppm. The star (*) represents the signal of solvent. Note that no discernible changes were observed upon UV irradiation for 10 min.

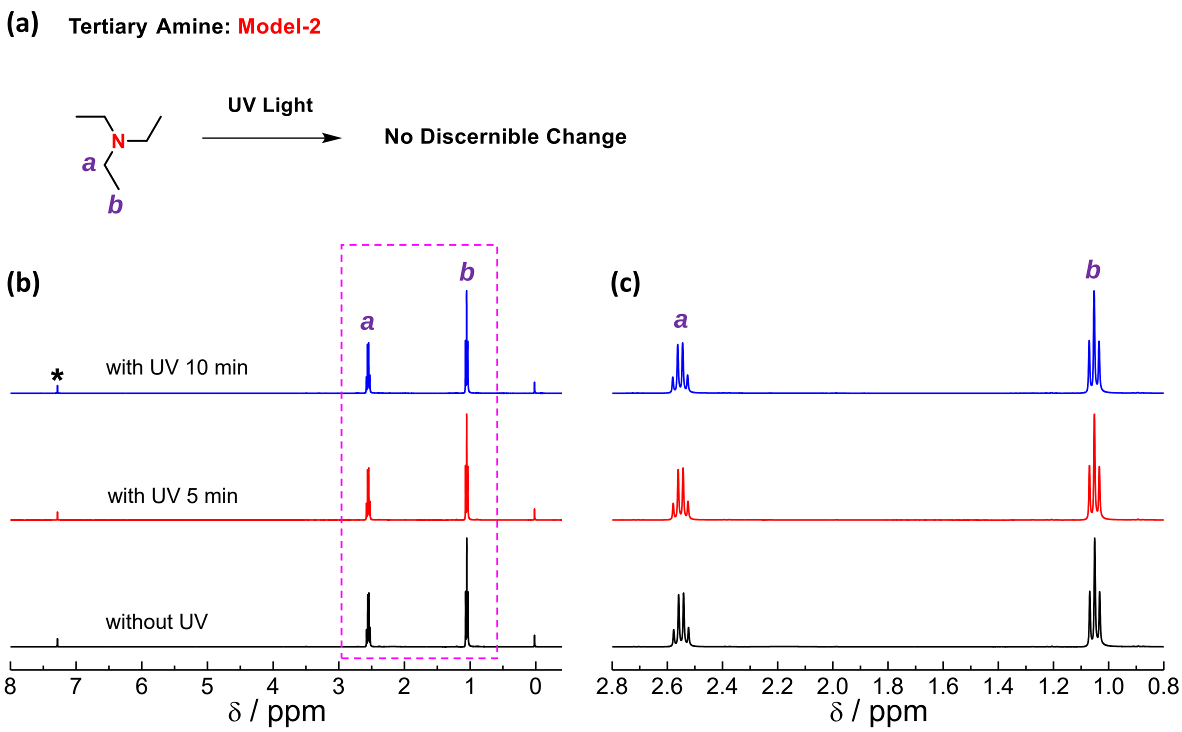

Supplementary Figure 24 (a) Schematics of photostability assay of triethylamine against UV light irradiation. (b) ^1H NMR spectra recorded in $CDCl_3$ for triethylamine without and with UV irradiation for 5 min and 10 min, respectively (UV light, 365 nm, 8 mW/cm^2). (c) Enlarged ^1H NMR spectra in the range of 0.8~2.8 ppm. The star (*) represents the signal of solvent. Note that no discernible changes were observed upon UV irradiation for 10 min.

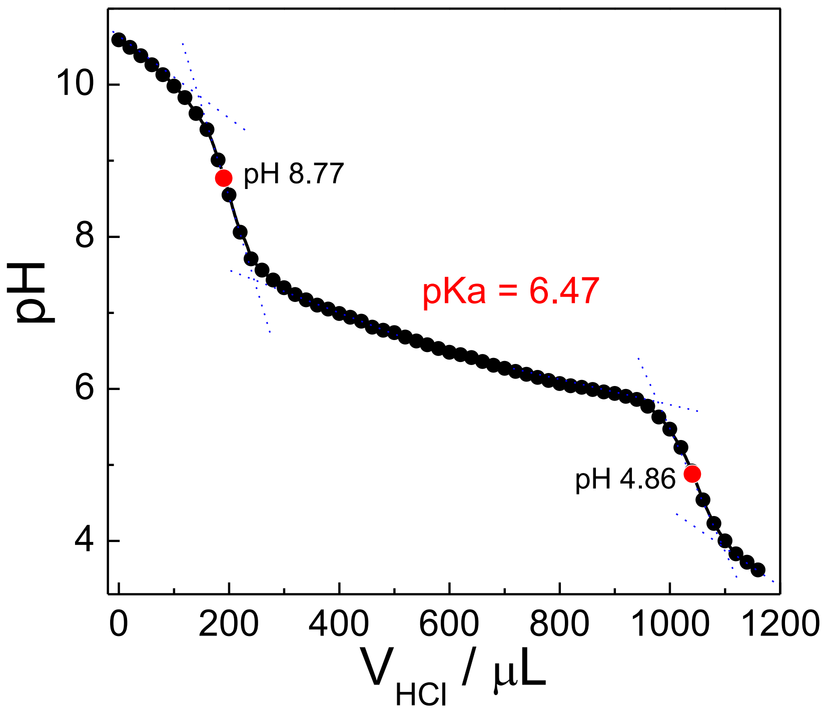

Supplementary Figure 25 Potentiometric titration curves recorded for PEO_{45}-*b*-PPA_{26} in aqueous media at 25 °C.

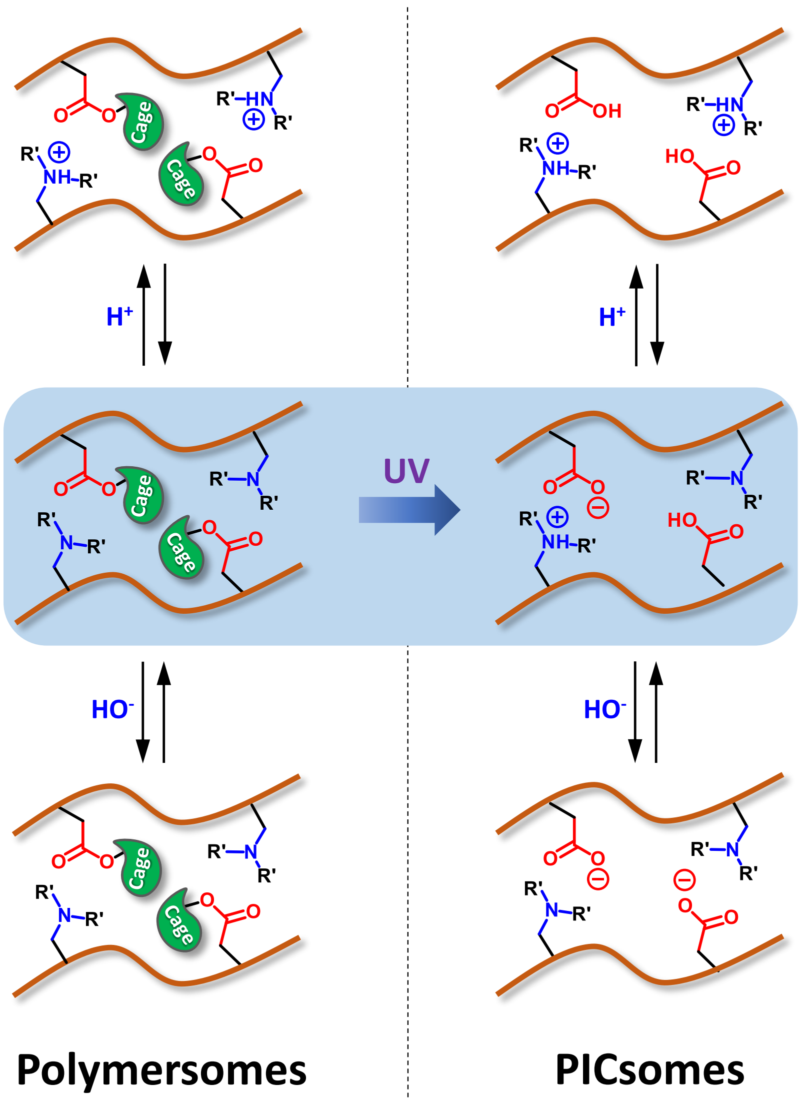

Supplementary Figure 26 Schematics of pH-triggered evolution of chemical structures within bilayers for PEO$_{45}$-*b*-P(NCMA$_{0.55}$-*co*-DPA$_{0.45}$)$_{29}$ polymersomes and corresponding PICsomes obtained after UV irradiation (0.1 g/L, Britton-Robinson buffer, pH 2-12, 12 mM, 25 °C).

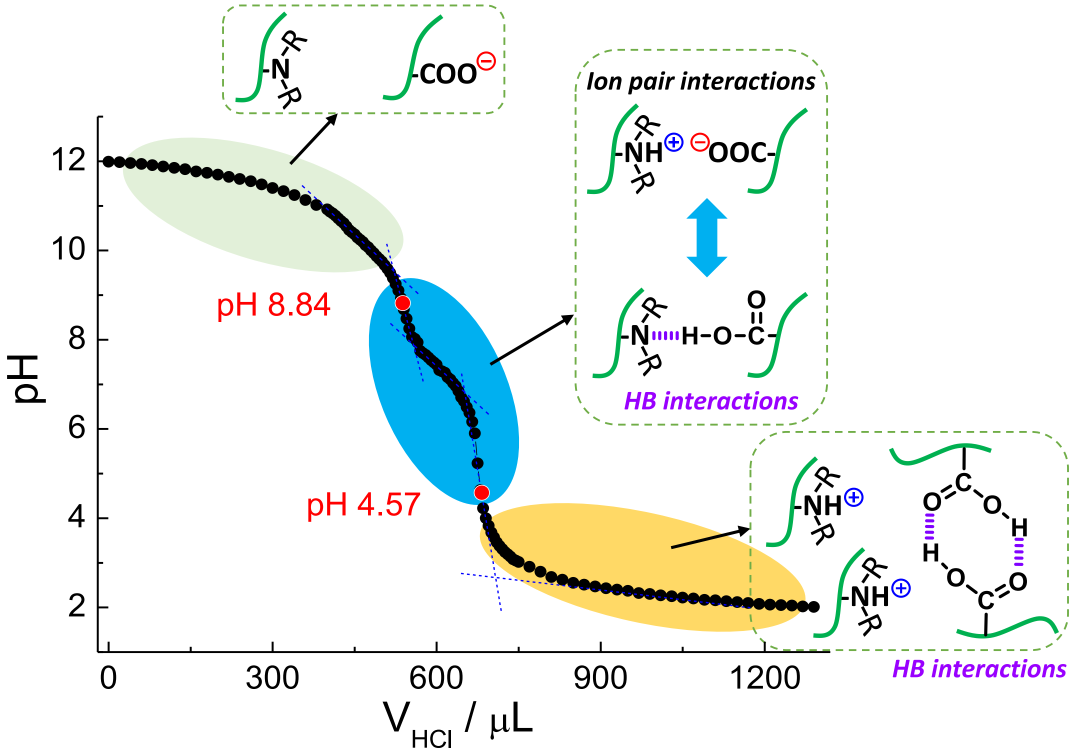

Supplementary Figure 27 Potentiometric titration curves recorded for the aqueous dispersion of PICsomes fabricated from PEO_{45}-*b*-P($NCMA_{0.55}$-*co*-$DPA_{0.45}$)$_{29}$ vesicles via UV irradiation (25 ^{o}C; 0.8 g/L, [NCMA] ~ 1.0 mM, [DPA] ~ 0.8 mM).

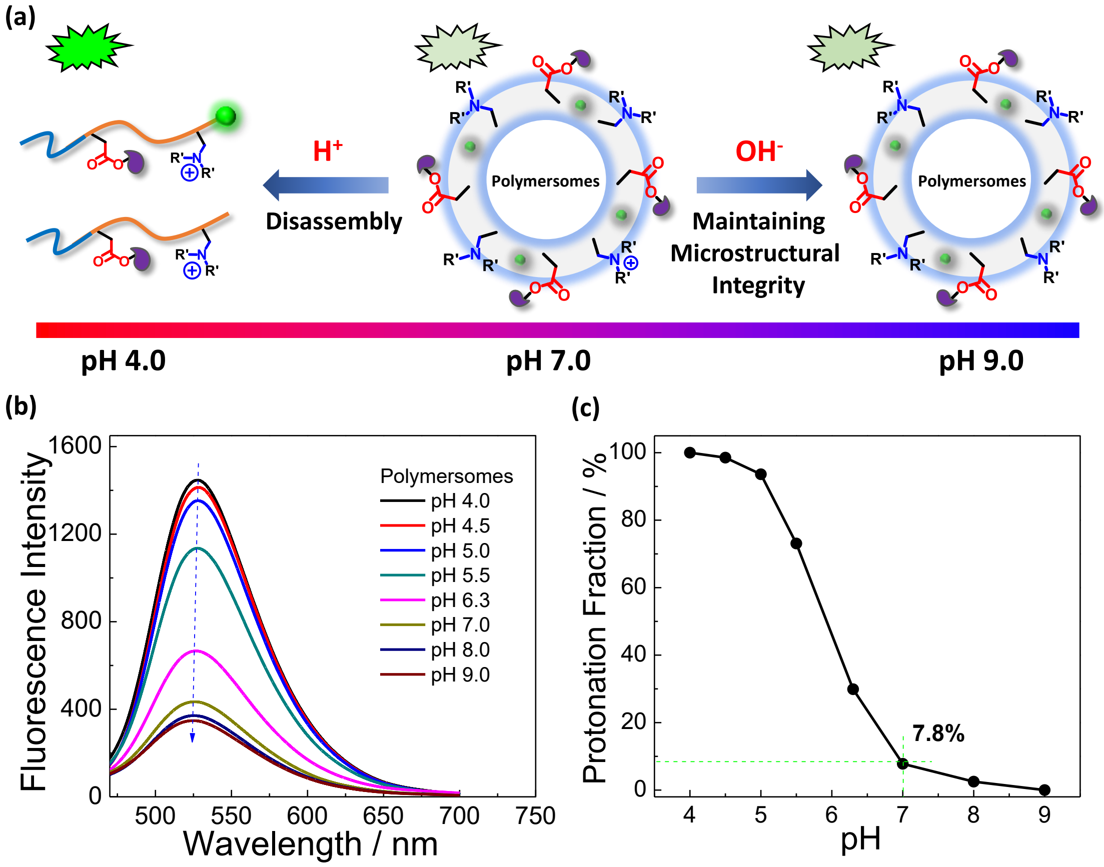

Supplementary Figure 28 (a) Schematics of pH-triggered microstructural evolution and concomitant changes in fluorescence emission for PEO-*b*-P(NCMA-*co*-DPA) polymersomes labeled with pH-sensitive naphthalimide-based fluorescent probe. Dye-labeled polymersomes were co-assembled from PEO_{45}-*b*-P($NCMA_{0.55}$-*co*-$DPA_{0.45}$)$_{29}$ and PEO_{45}-*b*-P($NCMA_{0.55}$-*co*-$DPA_{0.45}$)$_{29}$-*Naphthalimide* (8:2 molar ratio). (b,c) pH-dependent changes of fluorescence emission intensities and protonation degrees of tertiary amines determined for naphthalimide-labeled polymersomes in aqueous media. All experiments were conducted at a polymer concentration of 0.1 g/L ([NCMA] ~ 0.12 mM, [DPA] ~ 0.10 mM) in Britton-Robinson buffer (pH 4-9, 12 mM).

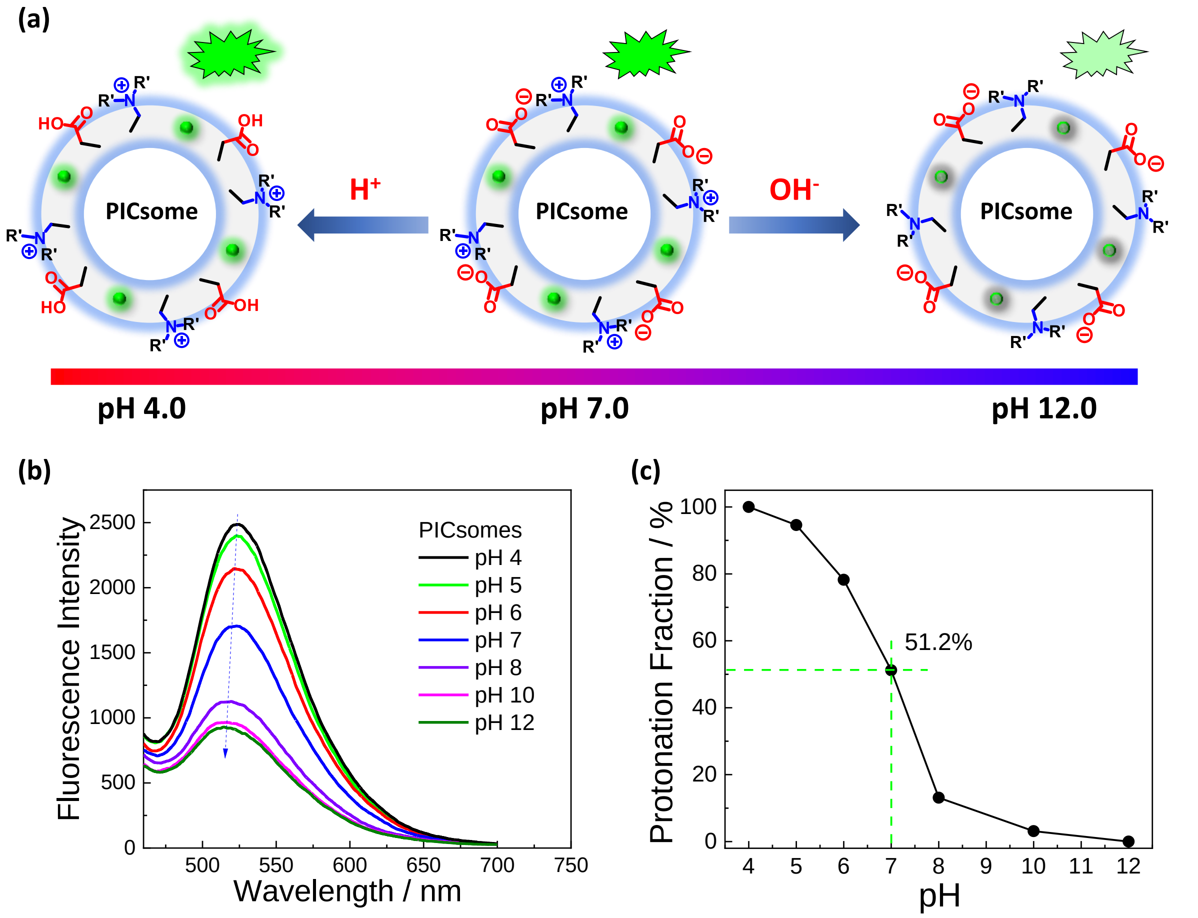

Supplementary Figure 29 (a) Schematics of pH-triggered microstructural evolution and concomitant changes in fluorescence emission for dye-labeled PICsomes fabricated from the co-assembly of PEO_{45}-*b*-P($NCMA_{0.55}$-*co*-$DPA_{0.45}$)$_{29}$ and PEO_{45}-*b*-P($NCMA_{0.55}$-*co*-$DPA_{0.45}$)$_{29}$-*Naphthalimide* (8:2 molar ratio) and subsequent UV irradiation for 10 min. (b,c) pH-dependent changes of fluorescence emission intensities and protonation degrees of tertiary amine moieties determined for naphthalimide-labeled PICsomes in aqueous media. All experiments were conducted at a polymer concentration of 0.1 g/L ([NCMA] ~ 0.12 mM, [DPA] ~ 0.10 mM) in Britton-Robinson buffer (pH 4-12, 12 mM).

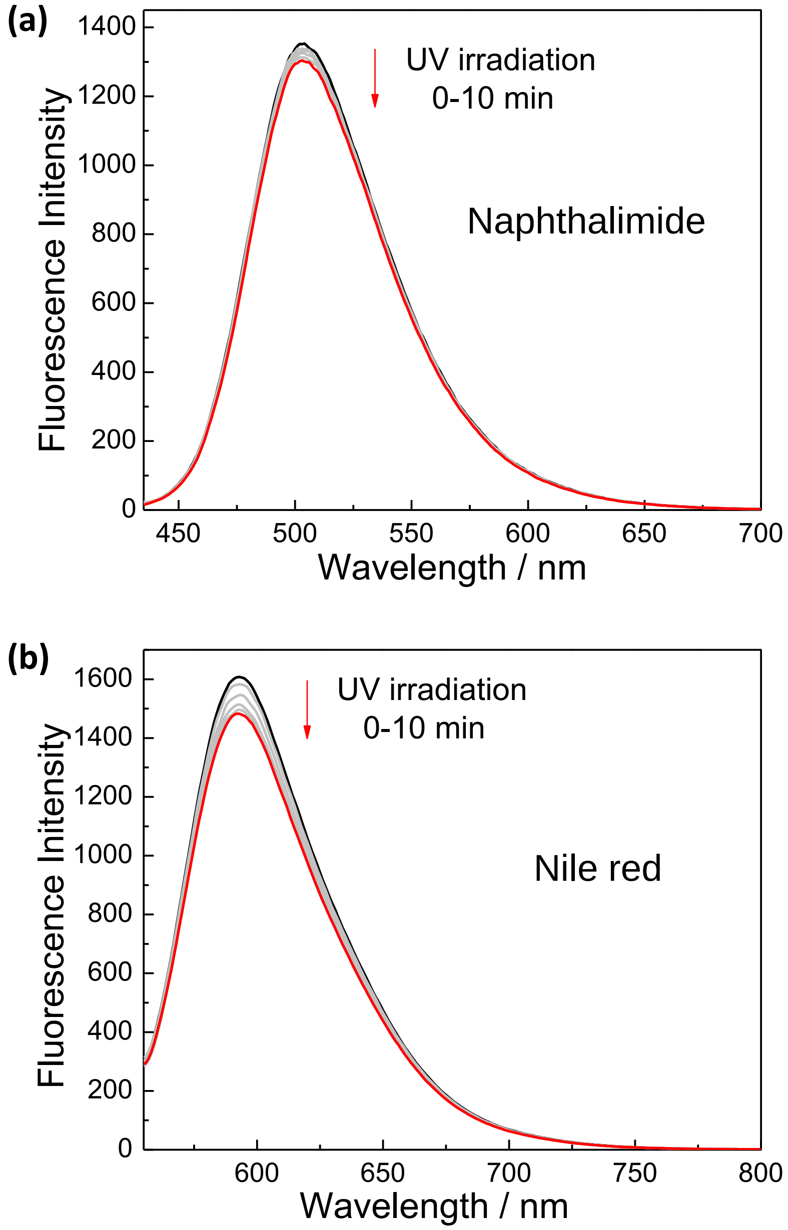

Supplementary Figure 30 UV irradiation duration-dependent changes of fluorescence emission intensities of (a) naphthalimide and (b) Nile red, respectively. All data were obtained at a dye concentration of 10 μM in THF (25 °C, UV irradiation for 0-10 min; emission spectra were recorded at 2 min intervals; 365 nm, 8 mW/cm^2).

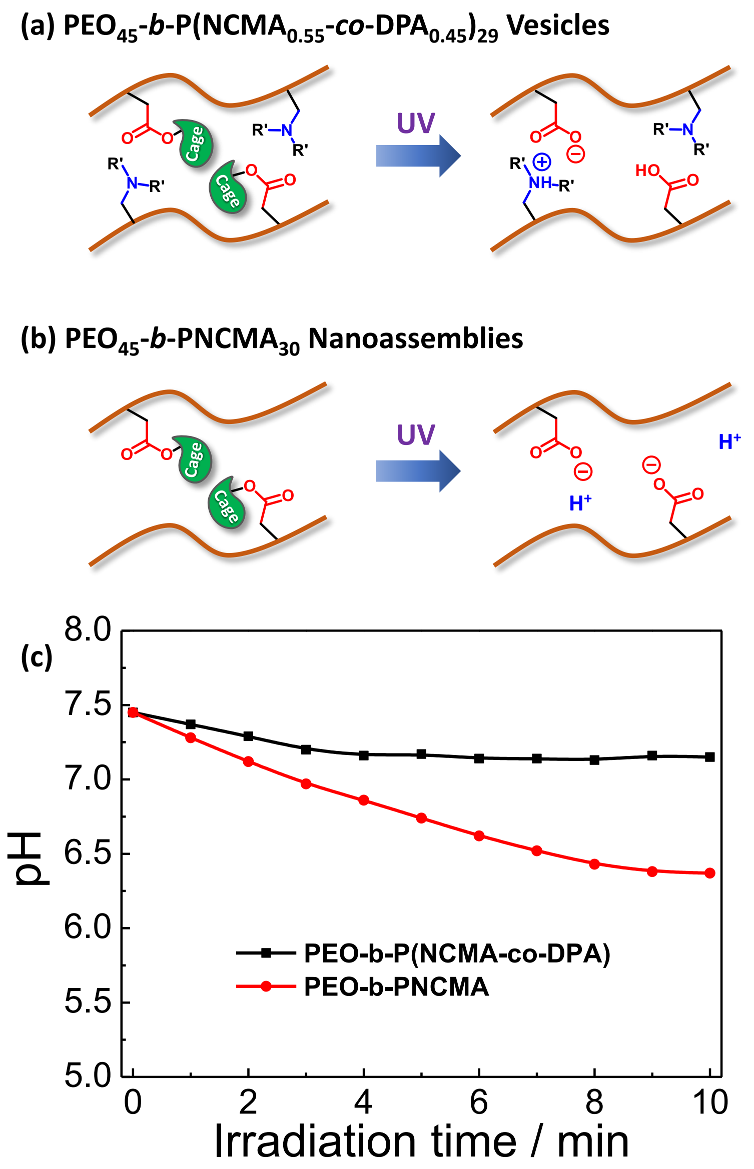

Supplementary Figure 31 Schematics of the extent of ionization of carboxyl moieties and ion-pair formation for (a) PEO_{45}-*b*-P($NCMA_{0.55}$-*co*-$DPA_{0.45}$)$_{29}$ polymersomes and (b) PEO_{45}-*b*-$PNCMA_{30}$ nanoassemblies upon UV irradiation. (c) The variation of pH with UV irradiation duration recorded for PEO_{45}-*b*-P($NCMA_{0.55}$-*co*-$DPA_{0.45}$)$_{29}$ polymersomes (0.1 g/L, [NCMA] ~ 0.12 mM, [DPA] ~ 0.10 mM) and PEO_{45}-*b*-$PNCMA_{30}$ nanoassemblies (0.06 g/L, [NCMA] ~ 0.12 mM) in non-buffered aqueous media.

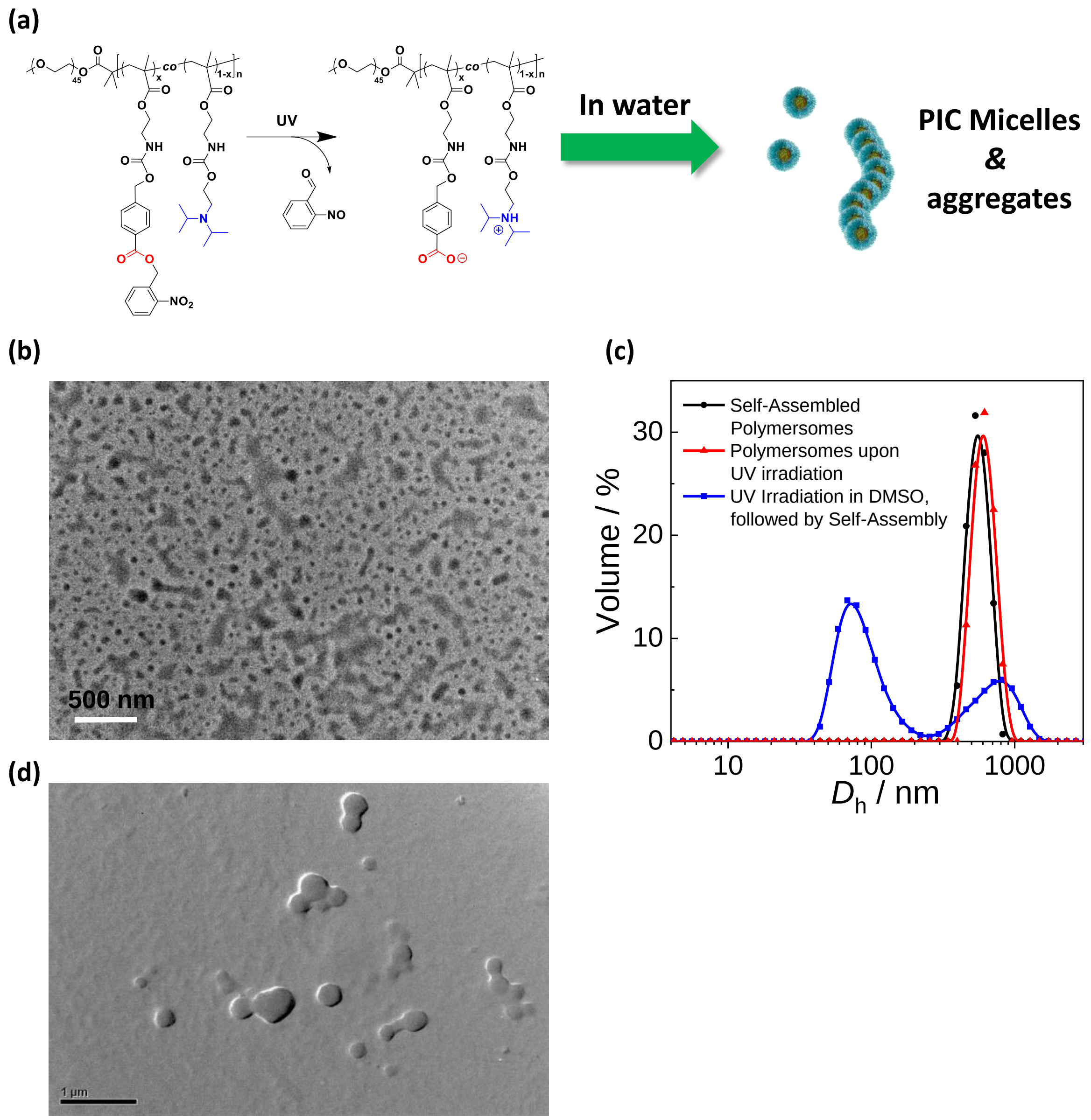

Supplementary Figure 32 (a) Schematic illustration of chemical structural changes of PEO_{45}-*b*-P($NCMA_{0.55}$-*co*-$DPA_{0.45}$)$_{29}$ upon UV irradiation, followed by self-assembly in aqueous media. (b) TEM characterization of nanoassemblies in neutral aqueous media formed by decaged PEO_{45}-*b*-P($NCMA_{0.55}$-*co*-$DPA_{0.45}$)$_{29}$ (obtained via UV irradiation in DMSO), revealing a mixture of aggregates with non-uniform sizes. (c) D_h distribution of PEO_{45}-*b*-P($NCMA_{0.55}$-*co*-$DPA_{0.45}$)$_{29}$ vesicles before and after UV irradiation in neutral aqueous media, and nanoassemblies of decaged PEO_{45}-*b*-P($NCMA_{0.55}$-*co*-$DPA_{0.45}$)$_{29}$. (d) TEM characterization of nanoassemblies formed by decaged PEO_{45}-*b*-P($NCMA_{0.55}$-*co*-$DPA_{0.45}$)$_{29}$ (obtained via UV irradiation in DMSO) in aqueous media at pH 2.

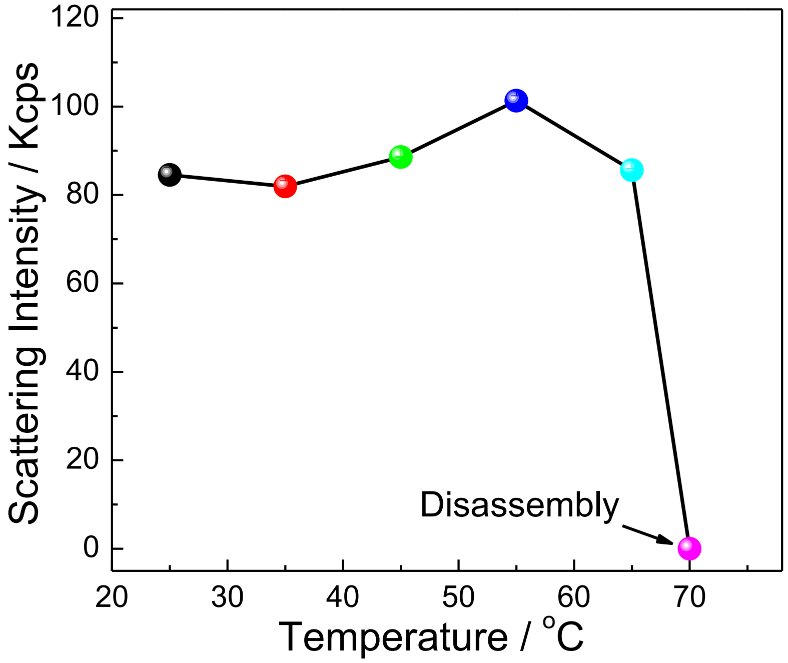

Supplementary Figure 33 Temperature-dependent scattered light intensities recorded for the aqueous dispersion of PICsomes (obtained from PEO_{45}-*b*-P($NCMA_{0.55}$-*co*-$DPA_{0.45}$)$_{29}$ polymersomes via UV irradiation) in the presence of 2.0 M NaCl. All data were obtained at a polymer concentration of 0.1 g/L in neutral aqueous media.

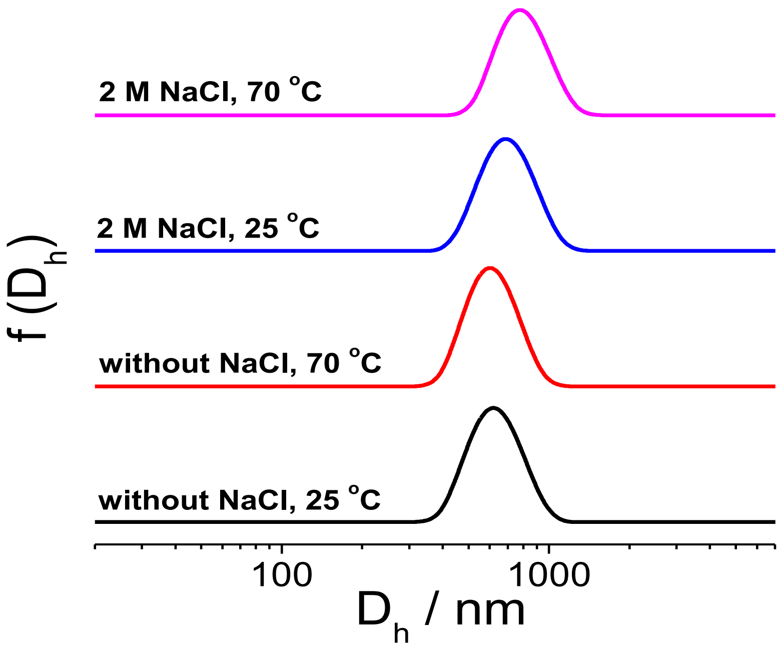

Supplementary Figure 34 The assay of microstructural stability of PEO_{45}-*b*-P($NCMA_{0.55}$-*co*-$DPA_{0.45}$)$_{29}$ polymersomes in aqueous media at different temperatures and ionic strengths. All experiments were conducted at a polymer concentration of 0.1 g/L in neutral aqueous media.

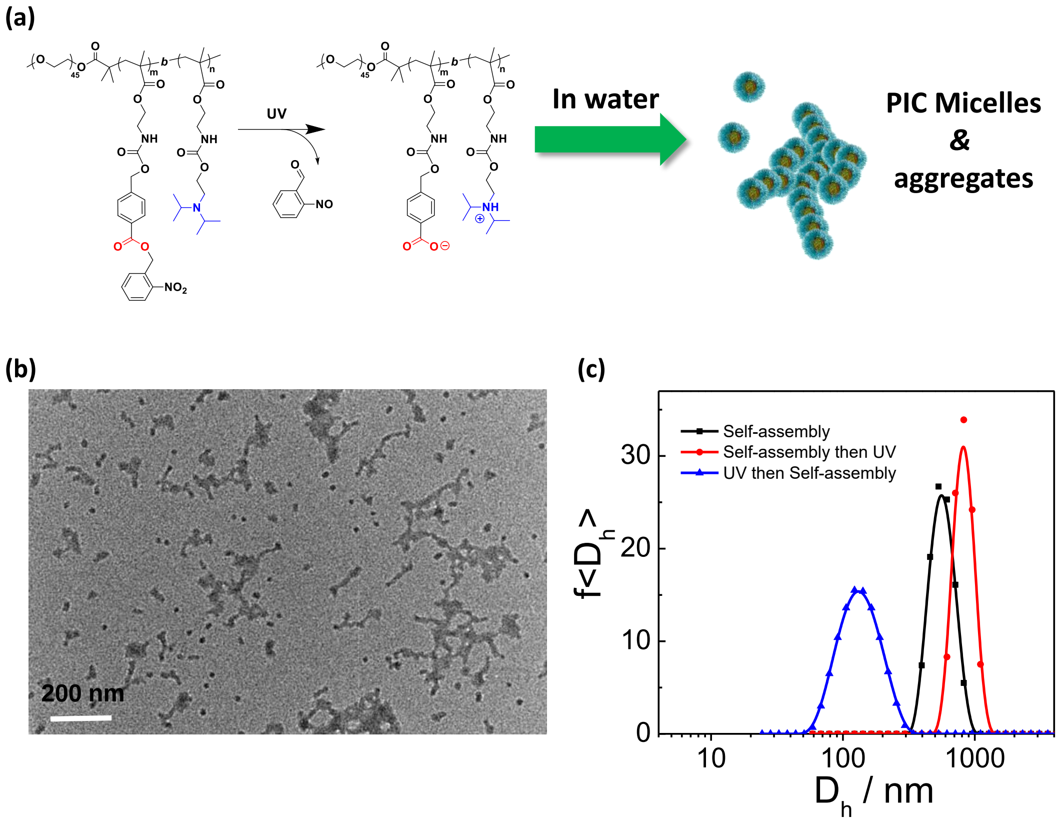

Supplementary Figure 35 (a) Schematic illustration of chemical structural changes of PEO_{45}-*b*-$PNCMA_{17}$-*b*-$PDPA_{21}$ triblock copolymer upon UV irradiation, followed by self-assembly in aqueous media. (b) TEM characterization of nanoassemblies of decaged PEO_{45}-*b*-$PNCMA_{17}$-*b*-$PDPA_{21}$ (obtained via UV irradiation in DMSO) in neutral aqueous media, revealing the formation of a mixture of aggregates with non-uniform sizes. (c) D_h distribution of PEO_{45}-*b*-$PNCMA_{17}$-*b*-$PDPA_{21}$ vesicles before and after UV irradiation in neutral aqueous media, and nanoassemblies of decaged PEO_{45}-*b*-$PNCMA_{17}$-*b*-$PDPA_{21}$ in neutral aqueous media.

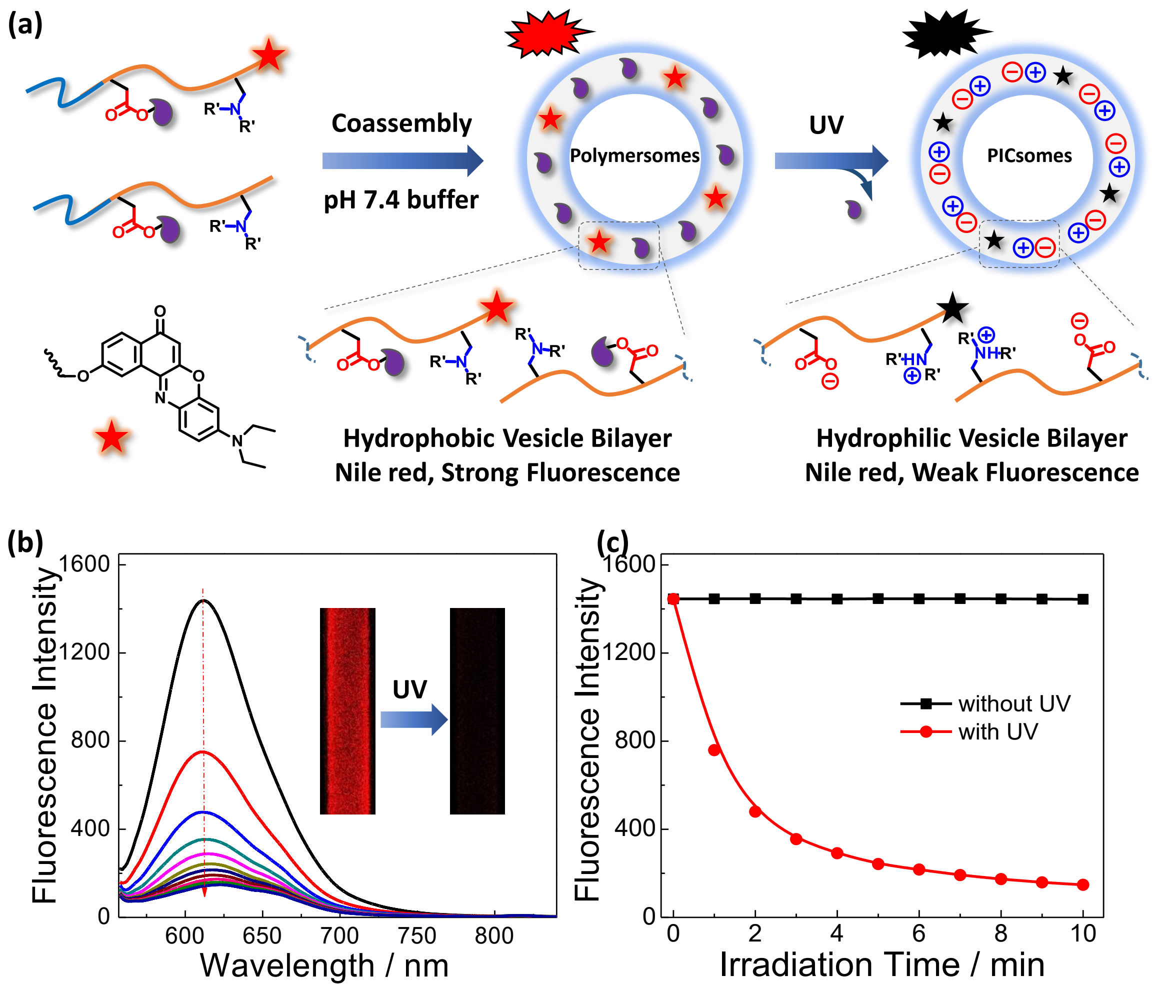

Supplementary Figure 36 (a) Schematics of microstructural evolution, hydrophobic-to-hydrophilic transition, and mechanisms of concomitant fluorescence emission changes during polymersome- to-PICsome transition upon UV irradiation of Nile red-labeled vesicles co-assembled from PEO_{45}-*b*-P($NCMA_{0.55}$-*o*-$DPA_{0.45}$)$_{29}$ and PEO_{45}-*b*-P($NCMA_{0.55}$-*co*-$DPA_{0.45}$)$_{29}$-*Nile red* (8:2 molar ratio). (b,c) Irradiation duration-dependent changes in fluorescence emission intensities recorded for Nile red-labeled vesicles. The inset in (b) shows macroscopic images recorded under 365 nm UV irradiation for polymersomes and corresponding PICsomes, respectively. All experiments were conducted at a polymer concentration of 0.1 g/L in phosphate buffer (pH 7.4, 10 mM).

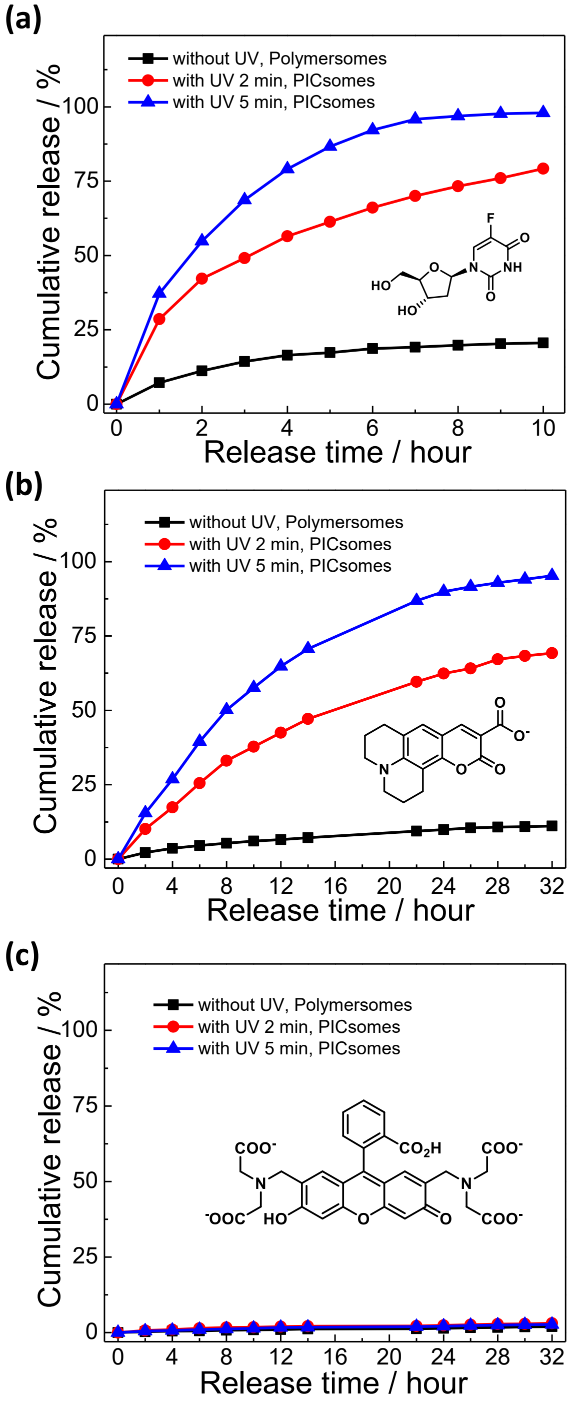

Supplementary Figure 37 Release profiles of (a) 5-Fu, (b) coumarin 343, and (c) calcein from aqueous interiors of PEO_{45}-*b*-P($NCMA_{0.55}$-*co*-$DPA_{0.45}$)$_{29}$ vesicles (without UV) and corresponding PICsomes (with UV, for 2 min or 5 min). All experiments were conducted at a polymer concentration of 0.1 g/L in phosphate buffer (pH 7.4, 10 mM, 37 ^{o}C).

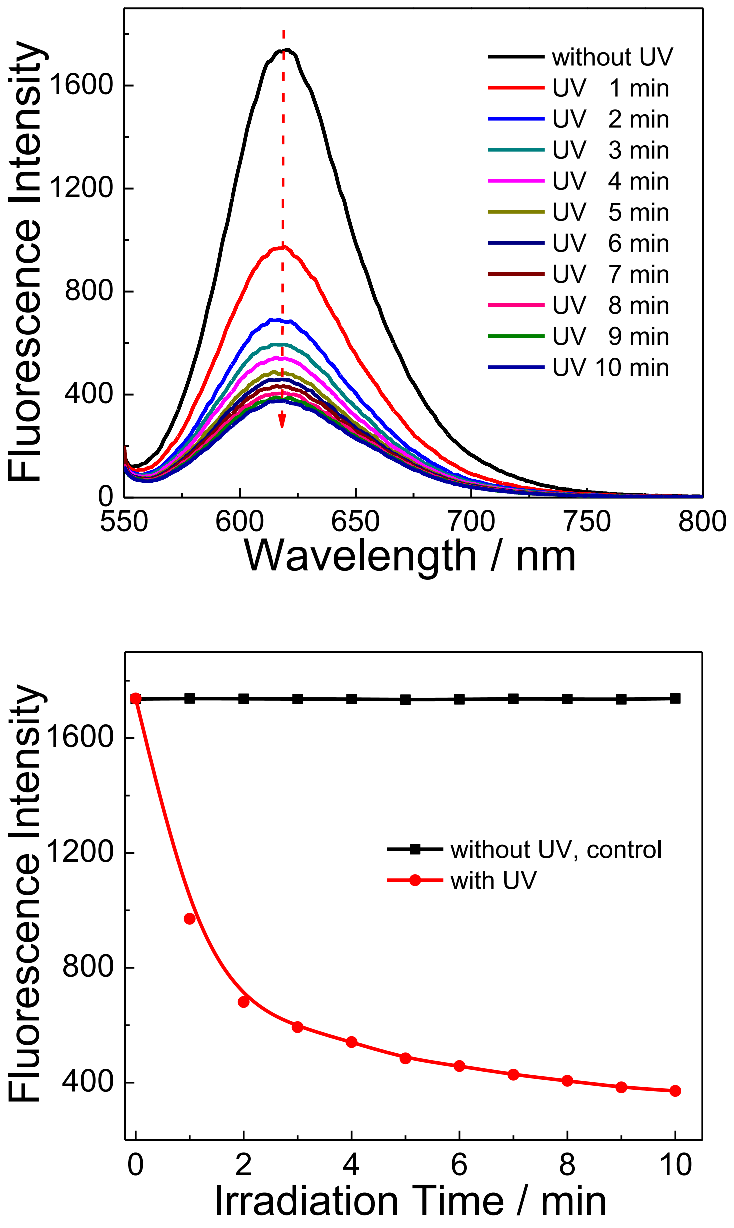

Supplementary Figure 38 UV irradiation time-dependent changes in fluorescence emission intensities recorded for PEO$_{45}$-*b*-PNCMA$_{17}$-*b*-PDPA$_{21}$ vesicles and corresponding PICsomes encapsulating Nile red probe within vesicle bilayers. All data were obtained at a polymer concentration of 0.1 g/L in phosphate buffer (pH 7.4, 10 mM).

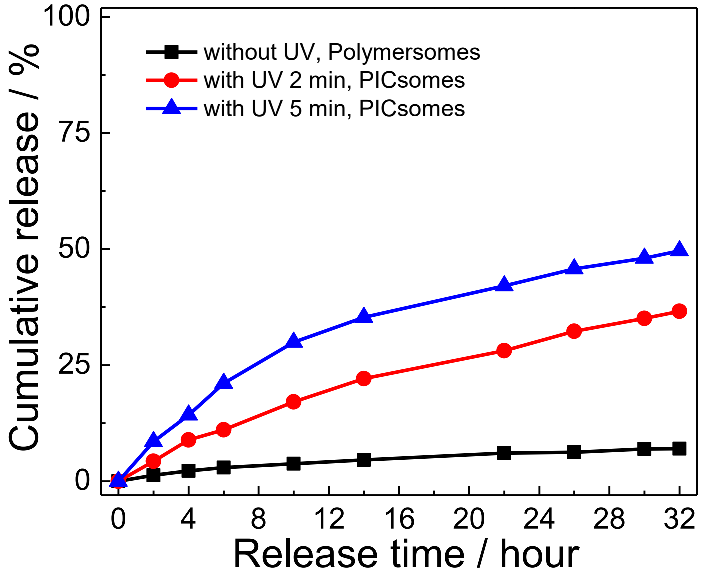

Supplementary Figure 39 Release profiles of loaded calcein from the aqueous interior of PEO_{45}-*b*-$PNCMA_{17}$-*b*-$PDPA_{21}$ vesicles (without UV) and corresponding PICsomes fabricated via UV irradiation (with UV, for 2 min or 5 min). All experiments were conducted at a polymer concentration of 0.1 g/L in phosphate buffer (pH 7.4, 10 mM, 37 ^{o}C).

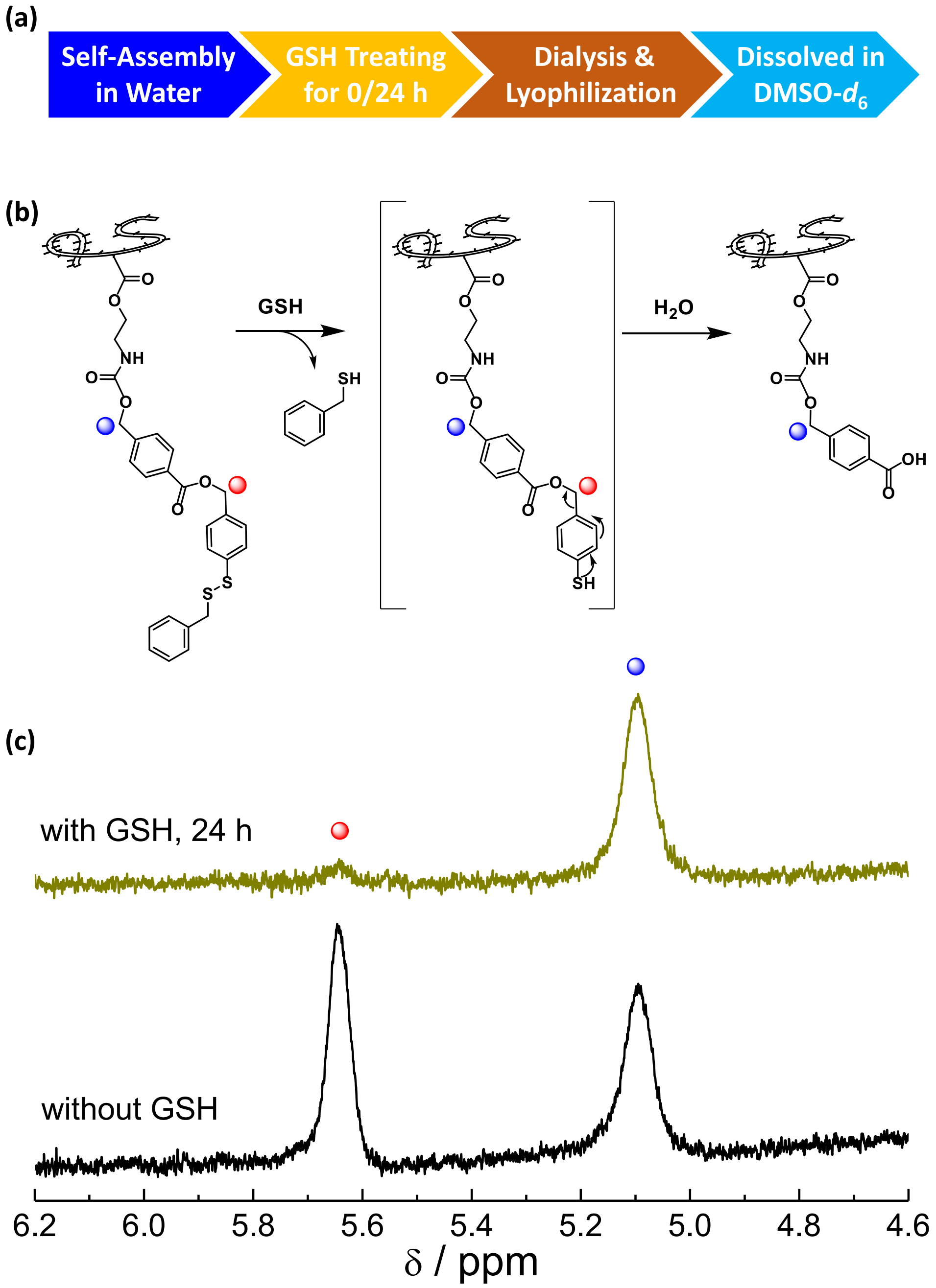

Supplementary Figure 40 ^{1}H NMR characterization of GSH-triggered carboxyl decaging process for PEO$_{45}$-*b*-P(DCMA$_{0.45}$-*co*-DPA$_{0.55}$)$_{33}$ polymersomes. (a) Brief illustration of experimental procedures. (b) Schematic illustration of chemical structural changes of DCMA moieties upon treating with GSH. (c) ^{1}H NMR spectra recorded for PEO$_{45}$-*b*-P(DCMA$_{0.45}$-*co*-DPA$_{0.55}$)$_{33}$ polymersomes before and after GSH treatment for 24 h, revealing an extent of carboxyl decaging of >99%. All data were obtained at a polymersome concentration of 0.1 g/L, [GSH] = 10 mM.

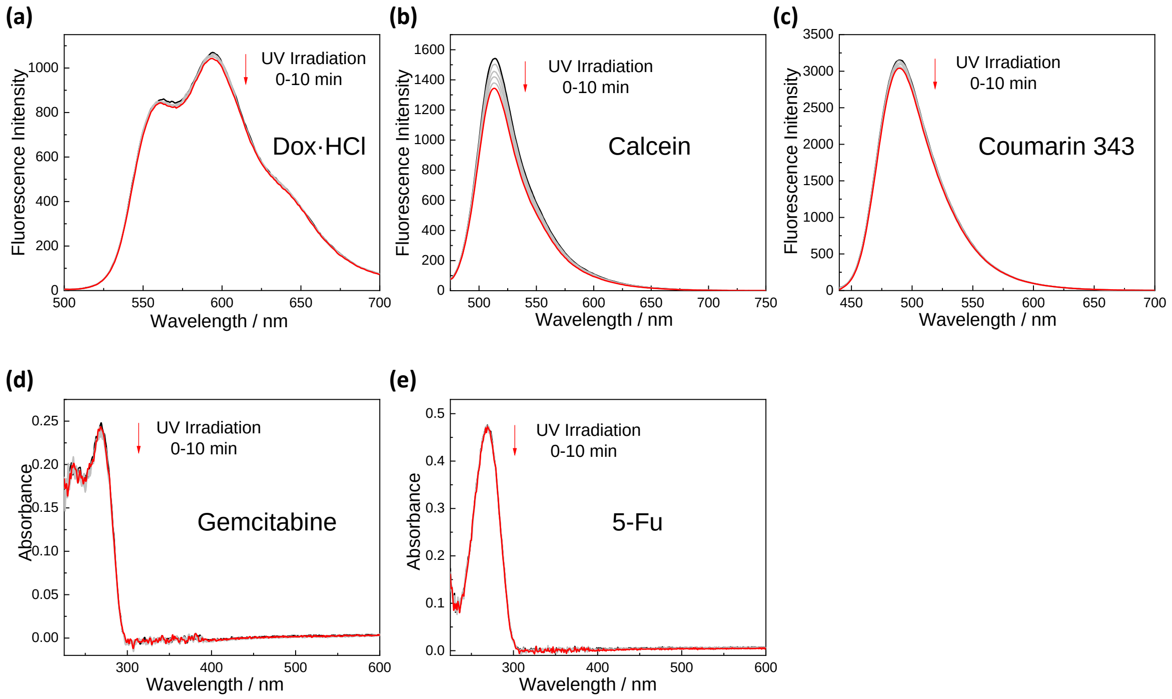

Supplementary Figure 41 (a,b,c) UV irradiation duration-dependent changes of fluorescence emission spectra recorded for (a) Dox ·HCl, (b) Calcein, and (c) Coumarin 343, respectively. (d,e) UV irradiation duration-dependent changes of UV-Vis absorption spectra recorded for (d) gemcitabine and (e) 5-Fu, respectively. All data were obtained at a drug or model drug concentration of 10 μM in phosphate buffer (10 mM, pH 7.4, 25 ^{o}C). UV irradiation for 0-10 min and the spectra were recorded at 2 min interval, 365 nm, 8 mW/cm^2.

References

[1] Lai, J. T.; Filla, D.; Shea, R. *Macromolecules* **2002**, *35*, 6754-6756.

[2] Rieger, J.; Stoffelbach, F.; Bui, C.; Alaimo, D.; Jerome, C.; Charleux, B. *Macromolecules* **2008**, *41*, 4065-4068.

[3] Zhu, K. N.; Liu, G. H.; Zhang, G. Y.; Hu, J. M.; Liu, S. Y. *Macromolecules* **2018**, *51*, 8530-8538.

[4] Shen, L.; Zhu, W.; Meng, X.; Guo, Z.; Tian, H. *Sci. China Ser. B-Chem.* **2009**, *52*, 821-826.
